# Current situation and progress toward the 2030 health-related Sustainable Development Goals in China: A systematic analysis

**DOI:** 10.1371/journal.pmed.1002975

**Published:** 2019-11-19

**Authors:** Shu Chen, Lei Guo, Zhan Wang, Wenhui Mao, Yanfeng Ge, Xiaohua Ying, Jing Fang, Qian Long, Qin Liu, Hao Xiang, Chenkai Wu, Chaowei Fu, Di Dong, Jiahui Zhang, Ju Sun, Lichun Tian, Limin Wang, Maigeng Zhou, Mei Zhang, Mengcen Qian, Wei Liu, Weixi Jiang, Wenmeng Feng, Xinying Zeng, Xiyu Ding, Xun Lei, Rachel Tolhurst, Ling Xu, Haidong Wang, Faye Ziegeweid, Scott Glenn, John S. Ji, Mary Story, Gavin Yamey, Shenglan Tang

**Affiliations:** 1 Global Health Research Center, Duke Kunshan University, Kunshan, Jiangsu, China; 2 Duke Global Health Institute, Duke University, Durham, North Carolina, United States of America; 3 Research Department of Social Development, Development Research Center, State Council of People's Republic China, Beijing, China; 4 School of Public Health, Fudan University, Shanghai, China; 5 Institute for Health Sciences, Kunming Medical University, Kunming, Yunnan, China; 6 School of Public Health and Management, Chongqing Medical University, Chongqing, China; 7 School of Health Sciences, Wuhan University, Wuhan, Hubei, China; 8 School of Political Science and Public Administration, Wuhan University, Wuhan, Hubei, China; 9 School of Public Health, Kunming Medical University, Kunming, Yunnan, China; 10 National Center for Chronic and Noncommunicable Disease Control and Prevention, Chinese Center for Disease Control and Prevention, Beijing, China; 11 Faculty of Clinical Sciences and International Public Health, Liverpool School of Tropical Medicine, Liverpool, United Kingdom; 12 Center of Health Human Resource Development, National Health Commission, Beijing, China; 13 Institute for Health Metrics and Evaluation, University of Washington, Seattle, Washington, United States of America; 14 Environment Research Center, Duke Kunshan University, Kunshan, Jiangsu, China; University College London, UNITED KINGDOM

## Abstract

**Background:**

The Sustainable Development Goals (SDGs), adopted by all United Nations (UN) member states in 2015, established a set of bold and ambitious health-related targets to achieve by 2030. Understanding China’s progress toward these targets is critical to improving population health for its 1.4 billion people.

**Methods and findings:**

We used estimates from the Global Burden of Disease (GBD) Study 2016, national surveys and surveillance data from China, and qualitative data. Twenty-eight of the 37 indicators included in the GBD Study 2016 were analyzed. We developed an attainment index of health-related SDGs, a scale of 0–100 based on the values of indicators. The projection model is adjusted based on the one developed by the GBD Study 2016 SDG collaborators.

We found that China has achieved several health-related SDG targets, including decreasing neonatal and under-5 mortality rates and the maternal mortality ratios and reducing wasting and stunting for children. However, China may only achieve 12 out of the 28 health-related SDG targets by 2030. The number of target indicators achieved varies among provinces and municipalities. In 2016, among the seven measured health domains, China performed best in child nutrition and maternal and child health and reproductive health, with the attainment index scores of 93.0 and 91.8, respectively, followed by noncommunicable diseases (NCDs) (69.4), road injuries (63.6), infectious diseases (63.0), environmental health (62.9), and universal health coverage (UHC) (54.4). There are daunting challenges to achieve the targets for child overweight, infectious diseases, NCD risk factors, and environmental exposure factors. China will also have a formidable challenge in achieving UHC, particularly in ensuring access to essential healthcare for all and providing adequate financial protection. The attainment index of child nutrition is projected to drop to 80.5 by 2025 because of worsening child overweight. The index of NCD risk factors is projected to drop to 38.8 by 2025. Regional disparities are substantial, with eastern provinces generally performing better than central and western provinces. Sex disparities are clear, with men at higher risk of excess mortality than women. The primary limitations of this study are the limited data availability and quality for several indicators and the adoption of "business-as-usual" projection methods.

**Conclusion:**

The study found that China has made good progress in improving population health, but challenges lie ahead. China has substantially improved the health of children and women and will continue to make good progress, although geographic disparities remain a great challenge. Meanwhile, China faced challenges in NCDs, mental health, and some infectious diseases. Poor control of health risk factors and worsening environmental threats have posed difficulties in further health improvement. Meanwhile, an inefficient health system is a barrier to tackling these challenges among such a rapidly aging population. The eastern provinces are predicted to perform better than the central and western provinces, and women are predicted to be more likely than men to achieve these targets by 2030. In order to make good progress, China must take a series of concerted actions, including more investments in public goods and services for health and redressing the intracountry inequities.

## Abstract 2

Shenglan Tang and colleagues report China's current situation and projected achievement of the health related sustainable development goals by 2030.

## Author summary

**Why was the study done?:**

- China has made impressive progress in the Millennium Development Goals (MDGs) era, which ended in 2015.
- Despite China’s success in achieving the MDGs, it faces formidable challenges in achieving many targets in the health-related Sustainable Development Goals (SDGs), which have a 2030 target date.
- Understanding China’s current situation and progress toward achieving these goals is critical to the development of practical solutions for improving population health for its 1.4 billion people in the SDG era.

**What did the researchers do and find?:**

- We conducted an analysis of progress toward the health-related SDGs using data from the Global Burden of Disease (GBD) Study 2016, national surveys, surveillance data from China, and qualitative study results.
- We developed an index of the attainment of the health-related SDGs, using a scale of 0–100 based on the absolute values of indicators.
- We adjusted the projection model developed by the GBD 2016 SDG collaborators to produce sex-specific estimates, stratified by province, and explore sex and regional disparities. Around 120 key informants were purposively approached for thematic interviews.
- This research found that China has achieved several health-related SDG targets, especially on child nutrition and maternal and child health and reproductive health. Nevertheless, China may only achieve 12 out of the 28 selected health-related SDG targets by 2030.
- There are daunting challenges ahead for China in achieving the targets for child overweight, infectious diseases, noncommunicable disease risk factors, environmental exposure factors, and universal health coverage.
- Regional disparities were substantial, with eastern coastal provinces generally performing better than central and western provinces in most of the indicators. Sex disparities were clear, with men at higher risk of excess mortality than women.

**What do these findings mean?:**

- To achieve the targets of the health-related SDGs, China must take a series of concerted actions, including increased investment in public goods and services for health, addressing the regional inequities, and tackling the emerging new health challenges to ensure that no one is left behind.
- In those areas where China has already achieved the SDG targets, the government should synthesize the experiences and good practices that can be applied to the regions/provinces that need to catch up to the level of health development witnessed in developed regions/provinces.
- The Chinese government needs to examine the root causes of the challenges in achieving the health-related SDGs and develop an action plan to address these causes. The plan should prioritize key policy interventions for target populations and mobilize adequate resources to tackle these health challenges.
- It is important to address common problems by tackling cross-cutting issues in advancing the health-related SDGs, such as strengthening the enforcement of laws and regulations, establishing effective organizational structures, and human resources development.

## Introduction

China has achieved almost all of the Millennium Development Goals (MDGs) [1], including substantial reductions in extreme poverty, increased access to primary and secondary education, and improved environmental sustainability and gender equality. On three health-related MDGs, China exceeded average progress globally between 1990 and 2015 [2]. This includes an average reduction in neonatal, infant, and under-five mortality of over 80% and a reduction of 74.9% in maternal mortality [3]. HIV/AIDS prevalence has remained low, with over 80% of eligible patients receiving antiretroviral treatment [1]. New cases of tuberculosis (TB) and malaria have continued to decline [3]. The key drivers of success in achieving the MDGs in China include economic growth, increased public investment in health, the development of health-related laws and regulations, the establishment of state-supported health insurance schemes to increase population coverage, and multisectoral coordination and collaboration domestically and internationally.

Building on the success of the MDGs that ended in 2015, the United Nations (UN) Sustainable Development Goals (SDGs) constitute the new development agenda until 2030. Of the 17 SDG goals, only goal 3, “ensure healthy lives and promote well-being for all at all ages,” explicitly addresses health, although 10 other goals involve health-related indicators [4,5]. To monitor and evaluate the progress of member states, the Inter-Agency and Expert Group on SDG Indicators announced a total of 232 monitoring indicators, 50 of which are health related [6]. A number of initiatives have been launched to track global progress toward health-related SDGs, including Monitoring Health for the SDGs [7], Atlas of the SDGs [8], Index and Dashboards [9], and Global Burden of Disease (GBD) Study [10].

China faces formidable challenges in achieving the health-related SDGs. A recent analysis of the GBD Study 2017 found that China ranked 88th among the 195 countries and territories evaluated based on the health-related SDG index, an overall measure of the 41 health-related SDG indicators (the definition of each indicator can be found in another article published elsewhere) [10]. China scored 62 in the health-related SDG index, slightly higher than the global median index of 59.4 but much lower than the top three countries, which all scored ≥83.0. China performed especially poorly in hepatitis B incidence, air pollution mortality, poisoning mortality, and mean particulate matter (PM) 2.5, with the indices all below 40.

China faces unique health challenges in addition to those common to middle-income countries. These include the highest hepatitis burden in the world, accounting for one-third of the 240 million people living with chronic hepatitis B [11], and lacking an effective, comprehensive hepatitis disease control program. More than 300 million smokers live in China, around one-third of the world’s total, yet limited prevention measures exist [12]. China accounted for around 35% of global deaths due to ambient air pollution in 2013 but faces substantial challenges to improve air quality [13]. There are further significant challenges to reducing regional social and health disparities in the context of sluggish economic growth, enlarging income gaps, an aging population, and urbanization [14].

The Chinese government has made a strong political commitment to achieving the health-related SDG targets, particularly by issuing the Healthy China 2030 Planning Outline (Healthy China 2030) in 2016 by the State Council [15]. To support the achievement of these targets over the next 12 years, this study assesses the current situation of key health-related SDG indicators, grouped under different health topics, and analyzes the projected attainment of these indicators by the target year. The analysis examines regional and sex disparities whenever possible. Implications of these key findings for China and globally are discussed.

## Methods

### Sources of data

We collected quantitative data from governmental agencies and institutions of the National Health Commission of China [3]. In addition, we reviewed the literature, policy documents, and national survey reports to obtain data of key health indicators [16,17,18,19,20,21,22]. We also used data from the GBD Study 2016, including (1) estimates of morbidity, mortality, and prevalence of risk factors and their attributed morbidity and mortality from 1990–2016, stratified by age and sex, at national and provincial levels (data of Taiwan Province were not included because this paper focused on mainland China) and (2) estimates of health-related SDG monitoring indicators from 1990 to 2016 and projected values from 2017 to 2030 at national and provincial levels. Details of the GBD Study 2016 and its methodology have been reported elsewhere [23]. We did not use GBD Study 2017 results, because of lack of data availability at the time of conducting this study, and GBD Study 2016 results could provide results of the first year of the SDG era.

We also purposively interviewed about 120 key stakeholders with knowledge and expertise in relevant health areas of the SDGs at the national level and in selected provinces, including government officials, CDC health professionals, academic experts, and physicians. Semistructured interviews were conducted to qualitatively understand the current situation, gaps, and challenges in achieving the health-related SDG targets, particularly on topics of child nutrition, maternal and child health and reproductive health (MCHRH), TB, and noncommunicable diseases (NCDs). We obtained verbal informed consent from participants and anonymized their responses. Thematic analysis was applied to identify emerging themes. See S1 Appendix for details on the collection and analysis of qualitative data. This study only obtained ethical approval from the Duke University Institutional Review Board (IRB number: E0097).

### SDG indicators inclusion, categorization, and measurement

We selected 28 key health-related SDG indicators from SDGs 2, 3, 5, 6, 7, 8, and 11 out of the 37 indicators included in the GBD Study 2016. The GBD Study 2016 excluded 13 indicators from 50 health-related SDG indicators because of poor data availability among all nations across years and ongoing and planned analysis of these indicators. General inclusion criteria of our study were (1) direct relevance to health improvement in the Chinese context, (2) clear target value defined by the SDGs or international guidelines/documents from, e.g., World Health Organization (WHO), (3) availability of reliable data in China, and (4) potential impacts on health policy development in the near future. We excluded nine indicators: adolescent birth rate, intimate partner violence, disease burden attributable to occupational risks, disaster mortality, homicide, conflict and terrorism mortality, violence prevalence, childhood sexual abuse, and well-certified death registration. Detailed reasons why these nine indicators were excluded are in S1 Appendix.

The 28 indicators were further categorized into seven thematic topics: (1) child nutrition, (2) MCHRH, (3) infectious diseases, (4) NCDs and mental health, (5) road injuries, (6) environmental health, and (7) universal health coverage (UHC) (Table 1). NCDs and environmental health were subcategorized into risk factors and outcomes.

**Table 1:** The selected 28 SDG monitoring indicators and their targets.

Thematic topics	Health-related SDG indicator	Indicator description	Target source	Target description	Target year
Child nutrition	2.2.1	Prevalence of stunting in children under 5 years old (%)	Global Nutrition Targets 2025	Achieve a 40% reduction in the number of children under 5 years old who are stunted.	2025
	2.2.2a	Prevalence of wasting in children under 5 years old (%)	Global Nutrition Targets 2025	Reduce and maintain childhood wasting to less than 5%.	2025
	2.2.2b	Prevalence of overweight in children aged 2–4 years (%)	Global Nutrition Targets 2025	Ensure that there is no increase in childhood overweight.	2025
MCHRH	3.1.1	Maternal mortality ratio (maternal deaths per 100,000 live births) in women aged 10–54 years	SDG	By 2030, reduce the global maternal mortality ratio to less than 70 per 100,000 live births.	2030
	3.1.2	Proportion of births attended by skilled health personnel (%)	No		2030
	3.2.1	Under-5 mortality rate (probability of dying before the age of 5 per 1,000 live births)	SDG	By 2030, end preventable deaths of newborns and children under 5 years of age, with all countries aiming to reduce neonatal mortality to at least as low as 12 per 1,000 live births and under-5 mortality to at least as low as 25 per 1,000 live births.	2030
	3.2.2	Neonatal mortality rate (probability of dying during the first 28 days of life per 1,000 live births)	SDG	By 2030, end preventable deaths of newborns and children under 5 years of age, with all countries aiming to reduce neonatal mortality to at least as low as 12 per 1,000 live births and under-5 mortality to at least as low as 25 per 1,000 live births.	2030
	3.b.1	Geometric mean of the coverage of eight vaccines, conditional on inclusion in national vaccine schedules, in target populations (%)	SDG	Support the research and development of vaccines and medicines for the communicable and NCDs that primarily affect developing countries, provide access to affordable essential medicines and vaccines, in accordance with the Doha Declaration on the TRIPS Agreement and Public Health, which affirms the right of developing countries to use the full provisions in the Agreement on Trade-Related Aspects of Intellectual Property Rights regarding flexibilities to protect public health, and, in particular, provide access to medicines for all.	2030
	3.7.1	Proportion of women of reproductive age (15–49 years) who have their need for family planning satisfied with modern methods (%)	SDG	By 2030, ensure universal access to sexual and reproductive healthcare services, including for family planning, information and education, and the integration of reproductive health into national strategies and programs.	2030
Infectious diseases	3.3.1	Age-standardized rate of new HIV infections (per 1,000 population)	Accelerating progress on HIV, TB, malaria, hepatitis, and NTDs	Reduce new HIV infections to less than 500,000 by 2020 (compared with 2.1 million new HIV infections in 2010) and, by 2030, reduce the annual number of new infections by 90%.	2030
	3.3.2	Age-standardized rate of TB cases (per 100,000 population)	Accelerating progress on HIV, TB, malaria, hepatitis, and NTDs	Reduce TB incidence rate by 80% by 2030 (compared with 2015).	2030
	3.3.3	Age-standardized rate of malaria cases (per 1,000 population)	Global technical strategy for malaria, 2016–2030	Reduce malaria case incidence by at least 90% by 2030 (compared with 2015).	2030
	3.3.4	Age-standardized rate of hepatitis B incidence (per 100,000 population)	Global health sector strategy on viral hepatitis, 2016–2021	Between 6 and 10 million infections are reduced to 0.9 million infections by 2030 (95% decline in hepatitis B virus infections, 80% decline in hepatitis C virus infections).	2030
	3.3.5	Age-standardized prevalence of the sum of 15 NTDs (%) *Prevalence estimates reported here may exceed 100% because they reflect the sum of prevalent cases of 15 NTDs	Accelerating progress on HIV, TB, malaria, hepatitis, and NTDs	The NTD community now has to operationalize its proposed indicator, deciding what “the end” of NTDs by 2030 means in terms of the number of people requiring interventions—globally as well as for individual countries and NTDs. Based on the projections mentioned above, a “90% reduction in the number of people requiring interventions” could be one milestone for success at the global level.	2030
NCDs and mental health	3.4.1	Age-standardized death rate due to cardiovascular disease, cancer, diabetes, and chronic respiratory disease in populations aged 30–70 (per 100,000 population)	SDG	By 2030, reduce by one-third premature mortality from NCDs through prevention and treatment and promote mental health and well-being.	2030
	3.4.2	Age-standardized death rate due to self-harm (per 100,000 population)	SDG	By 2030, reduce by one-third premature mortality from NCDs through prevention and treatment and promote mental health and well-being.	2030
	3.5.2	Risk-weighted prevalence of alcohol consumption, as measured by the SEV for alcohol use (%)	Global action plan for the prevention and control of NCDs, 2013–2020	At least 10% relative reduction in the harmful use of alcohol 2, as appropriate, within the national context.	2025
	3.a.1	Age-standardized prevalence of daily smoking in populations aged 10 years and older (%)	Global action plan for the prevention and control of NCDs, 2013–2020	A 30% relative reduction in prevalence of current tobacco use in persons aged 15+ years.	2025
Road injuries	3.6.1	Age-standardized death rate due to road injuries (per 100,000 population)	SDG	By 2020, halve the number of global deaths and injuries from road traffic accidents.	2020
Environmental health	3.9.1	Age-standardized death rate attributable to household air pollution and ambient air pollution (per 100,000 population)	SDG	By 2030, substantially reduce the number of deaths and illnesses from hazardous chemicals and air, water, and soil pollution and contamination.	2030
	3.9.2	Age-standardized death rate attributable to unsafe WaSH (per 100,000 population)	SDG	By 2030, substantially reduce the number of deaths and illnesses from hazardous chemicals and air, water, and soil pollution and contamination.	2030
	3.9.3	Age-standardized death rate due to unintentional poisonings (per 100,000 population)	SDG	By 2030, substantially reduce the number of deaths and illnesses from hazardous chemicals and air, water, and soil pollution and contamination.	2030
	6.1.1	Risk-weighted prevalence of populations using unsafe or unimproved water sources, as measured by the SEV for unsafe water (%)	SDG	By 2030, achieve universal and equitable access to safe and affordable drinking water for all.	2030
	6.2.1a	Risk-weighted prevalence of populations using unsafe or unimproved sanitation, as measured by the SEV for unsafe sanitation (%)	SDG	By 2030, achieve access to adequate and equitable sanitation and hygiene for all and end open defecation, paying special attention to the needs of women and girls and those in vulnerable situations.	2030
	6.2.1b	Risk-weighted prevalence of populations without access to a handwashing facility, as measured by the SEV for unsafe hygiene (%)	SDG	By 2030, achieve access to adequate and equitable sanitation and hygiene for all and end open defecation, paying special attention to the needs of women and girls and those in vulnerable situations.	2030
	7.1.2	Risk-weighted prevalence of household air pollution, as measured by the SEV for household air pollution (%)	SDG	By 2030, ensure universal access to affordable, reliable, and modern energy services.	2030
	11.6.2	Population-weighted mean levels of fine PM smaller than 2.5 μm in diameter (PM2.5)	WHO air quality guidelines for PM, ozone, nitrogen dioxide, and sulphur dioxide: global update 2005: summary of risk assessment	An annual average concentration of 10 μg/m^3^ was chosen as the long-term guideline value for PM2.5.	2030
UHC	3.8.1	Coverage of essential health services, as defined by the UHC index comprising the coverage of nine tracer interventions and risk-standardized death rates from 32 causes amenable to personal healthcare (scale of 0 to 100)	SDG	Achieve UHC, including financial risk protection; access to quality essential healthcare services; and access to safe, effective, quality, and affordable essential medicines and vaccines for all.	2030

Abbreviations: MCHRH, maternal and child health and reproductive health; NCD, noncommunicable disease; NTD, neglected tropical disease; SDG, Sustainable Development Goal; SEV, summary exposure value; PM, particulate matter; TB, tuberculosis; TRIPS, trade-related aspects of intellectual property rights; UHC, universal health coverage; WaSH, water, sanitation, and hygiene

To compare performance of these indicators and to combine the indicators in each thematic topic, we transformed the absolute values of the 28 indicators to an index ranging from 0 to 100. Among values estimated for the year 1990–2015 of each indicator in China, the worst value was set as 0 after we teased out the outliers (outliers were defined as values standing outside of 1.5 times the interquartile range). The scaling of attainment index for the indicators of incidence and mortality rates were performed in log space. The values of the SDG 2030 targets (some of which have a target year of 2020 or 2025 according to WHO guidelines; see Table 1) were set as 100. Details about how they were set for goals without quantitative targets can be found in S1 Appendix.

The index of each thematic topic was obtained through calculating the geometric mean of the index of each indicator under the same topic. We adjusted the score to 1 if it is less than 1 to calculate a valid geometric mean. We followed the previous studies and adopted the preference-weighted approach to assign equal weight to the indicators in accordance to the preferences of UN member states stated in the 2030 Agenda for Sustainable Development [10,23,24,25].

### SDG indicators projections

We projected the sex-specific absolute values for indicators whenever necessary. After comparing project results from five projection methods for accuracy and reliability, we adopted the modified projection model derived from the GBD 2016 SDG study [23].

The following adjustments were made based on the GBD 2016 SDG projection model: (1) we used weighted mean annual rate of change to predict the data trend after the weight matrix *ω* was confirmed, and (2) the *ω* was defined by the trend of each indicator in each province in China. Therefore, we only present the scaled mean scores without uncertainty intervals. Detailed methods about the projection model can be found in S1 Appendix.

### SDG indicators attainment and its progress

We put an attainment index of 90 as the threshold for the successful attainment of the health- related SDG targets after considering technical and political factors because we wanted to aim high for the Chinese policy makers of both national and provincial governments so that they can invest more resources and make greater efforts in improving the health of their people.

To further compare the progress of each thematic topic in each province, we calculated the annual change rate of the index from 2016 to 2030 by dividing the change of index by the number of years.

### Geographic and sex disparity

The analysis was, whenever possible, disaggregated by sex and geographic region. Geographic disparities were explored through the analysis of the seven topics at the provincial level. We also described regional disparities based on official statistical categorization of eastern, central, and western parts of China in line with the socioeconomic development status. Jiangsu, Hubei, and Yunnan provinces were selected to represent the high-, middle-, and low-income regions, respectively, for qualitative analysis. The three provinces were selected based on the location of our project partners, the feasibility of research implementation, and most importantly, the representativeness of the three different socioeconomic development levels. Rural/urban and sex disparities were also explored when data were available. To define accurately urban and rural populations in China has not been easy, but government agencies and institutions have increasingly used the actual location of people’s residence to define urban and rural populations, instead of the household registration (*hukou*) system in recent years. We acknowledge that sex differences refer to those that are based on biology or physiology, whereas gender differences go further to encompass socially constructed differences, but in our study, data were only available by sex and not gender.

### Data quality and methodology robustness

To ensure data quality of the GBD Study 2016 results, we compared the GBD Study 2016 data with other official sources of data in China, which reported the same indicators, including but not limited to national and provincial surveillance, survey, disease registry, and death registry [3,16,17,18,19,20,21,22]. In cases of significant inconsistencies, we consulted technical experts and the GBD Study 2016 team for clarifications of possible explanations and solutions.

To enhance the methodological robustness of the inclusion, categorization, measurement, and attainment of indicators, we organized consultation meetings with technical experts including from WHO and National Health Commission of China to reach practical solutions and consensus on the use of most reliable data.

## Results

### Overview of the current performance and future attainment of the health-related SDG indicators

Table 2 presents the national and provincial performance of the attainment index by each indicator and thematic topic in 2016. Overall, China performed best in child nutrition and MCHRH, with the attainment index scores of 93.0 and 91.8, respectively, in 2016, followed by NCDs (69.4), road injuries (63.6), infectious diseases (63.0), environmental health (62.9), and UHC (54.4). In looking further into environmental health and NCDs, the indicators related to the NCDs mortality (79.7) and environmental health mortality (75.5) scored higher. However, the indicators related to the NCD risk factors (60.5) and environmental health exposures (57.5) scored comparatively lower.

**Table 2:** The scores of the 28 selected SDG health indicators under different thematic topics of Chinese provinces in 2016.

		Child nutrition				Maternal and child health and reproductive health							Infectious diseases						NCD and mental health			NCD risk factors				Environmental outcomes				Environmental factors						
Provinces		Child stunting	Child wasting	Child overweight	Subtotal	Mat Mort ratio	Skilled birth attend	Under-5 Mort	Neonatal Mort	Vaccine Cov	FP Need Met, Mod	Subtotal	HIV Incid	TB Incid	Malaria Incid	Hep B Incid	NTD Prev	Subtotal	NCD Mort	Suicide Mort	Subtotal	Smoking Prev	Alcohol use	Subtotal	Road Inj Mort	Air Poll Mort	WaSH Mort	Poisoning Mort	Subtotal	Mean PM2.5	HH air Poll	Water	Sanitation	Hygiene	Subtotal	UHC index
	**China**	**86**	**100**	**94**	**91**	**100**	**92**	**100**	**100**	**98**	**66**	**92**	**40**	**63**	**100**	**49**	**80**	**63**	**72**	**89**	**80**	**74**	**50**	**60**	**64**	**58**	**95**	**79**	**75**	**31**	**76**	**56**	**72**	**66**	**55**	**54**
**Eastern China**	Beijing*	**92**	**100**	**0**	**30**	**100**	**96**	**100**	**100**	**79**	**79**	**92**	**91**	**83**	**100**	**62**	**89**	**84**	**81**	**97**	**88**	**69**	**0**	**8**	**86**	**72**	**99**	**96**	**88**	**5**	**96**	**70**	**95**	**81**	**41**	**71**
	Tianjin*	**91**	**100**	**0**	**30**	**100**	**96**	**100**	**100**	**81**	**76**	**92**	**89**	**87**	**100**	**60**	**88**	**84**	**76**	**95**	**85**	**70**	**0**	**8**	**74**	**63**	**98**	**88**	**82**	**0**	**93**	**67**	**93**	**79**	**27**	**67**
	**Hebei**	**87**	**100**	**94**	**92**	**100**	**91**	**100**	**100**	**93**	**66**	**91**	**93**	**84**	**100**	**53**	**87**	**82**	**69**	**91**	**79**	**78**	**40**	**55**	**50**	**55**	**97**	**71**	**72**	**12**	**72**	**58**	**77**	**70**	**44**	**57**
	**Liaoning**	**91**	**100**	**93**	**94**	**100**	**95**	**100**	**100**	**84**	**70**	**91**	**81**	**68**	**100**	**53**	**83**	**75**	**72**	**92**	**81**	**70**	**47**	**57**	**74**	**60**	**96**	**86**	**79**	**25**	**81**	**56**	**68**	**64**	**52**	**60**
	Shanghai*	**91**	**100**	**61**	**84**	**100**	**96**	**100**	**100**	**79**	**79**	**92**	**89**	**80**	**100**	**54**	**86**	**80**	**82**	**97**	**89**	**82**	**0**	**9**	**83**	**77**	**99**	**96**	**90**	**8**	**98**	**69**	**95**	**81**	**46**	**70**
	**Jiangsu**	**90**	**100**	**94**	**93**	**100**	**97**	**100**	**100**	**81**	**71**	**91**	**85**	**85**	**100**	**57**	**85**	**81**	**78**	**93**	**85**	**78**	**21**	**41**	**71**	**69**	**98**	**91**	**85**	**1**	**88**	**66**	**84**	**78**	**28**	**68**
	**Zhejiang**	**92**	**100**	**90**	**93**	**100**	**96**	**100**	**100**	**81**	**71**	**91**	**85**	**83**	**100**	**46**	**80**	**76**	**80**	**93**	**86**	**77**	**29**	**48**	**67**	**74**	**97**	**93**	**87**	**40**	**92**	**61**	**92**	**74**	**66**	**67**
	**Fujian**	**87**	**100**	**96**	**92**	**100**	**96**	**100**	**100**	**88**	**68**	**91**	**81**	**74**	**100**	**57**	**76**	**76**	**77**	**90**	**83**	**70**	**44**	**55**	**73**	**73**	**96**	**89**	**85**	**65**	**89**	**59**	**79**	**71**	**71**	**61**
	**Shandong**	**87**	**100**	**0**	**29**	**100**	**96**	**100**	**100**	**86**	**70**	**91**	**94**	**87**	**100**	**54**	**85**	**82**	**73**	**86**	**79**	**76**	**39**	**54**	**60**	**58**	**97**	**76**	**75**	**11**	**78**	**54**	**66**	**66**	**42**	**61**
	**Guangdong**	**89**	**100**	**95**	**93**	**100**	**96**	**100**	**100**	**85**	**73**	**92**	**44**	**62**	**100**	**49**	**69**	**62**	**76**	**94**	**85**	**71**	**28**	**45**	**78**	**69**	**97**	**91**	**85**	**52**	**90**	**64**	**89**	**76**	**71**	**61**
	**Hainan**	**87**	**100**	**99**	**93**	**100**	**94**	**100**	**100**	**97**	**65**	**92**	**65**	**30**	**100**	**44**	**77**	**58**	**72**	**87**	**79**	**61**	**51**	**56**	**70**	**62**	**94**	**79**	**77**	**70**	**75**	**57**	**74**	**68**	**68**	**51**
**Central China**	**Shanxi**	**87**	**100**	**94**	**92**	**100**	**82**	**100**	**100**	**93**	**65**	**89**	**83**	**79**	**100**	**50**	**80**	**77**	**70**	**93**	**81**	**72**	**53**	**62**	**55**	**55**	**96**	**70**	**72**	**40**	**68**	**57**	**75**	**69**	**58**	**56**
	**Jilin**	**87**	**100**	**95**	**92**	**100**	**95**	**100**	**100**	**91**	**67**	**91**	**81**	**58**	**100**	**49**	**84**	**72**	**69**	**92**	**79**	**72**	**50**	**60**	**75**	**54**	**96**	**77**	**74**	**30**	**71**	**58**	**76**	**70**	**55**	**56**
	**Heilongjiang**	**90**	**100**	**97**	**94**	**100**	**95**	**100**	**100**	**90**	**66**	**91**	**89**	**65**	**100**	**52**	**87**	**77**	**65**	**91**	**77**	**63**	**53**	**58**	**70**	**51**	**93**	**85**	**74**	**39**	**72**	**39**	**53**	**41**	**48**	**58**
	**Anhui**	**83**	**100**	**96**	**90**	**100**	**92**	**100**	**100**	**93**	**57**	**89**	**83**	**73**	**100**	**51**	**81**	**76**	**69**	**78**	**73**	**73**	**61**	**67**	**52**	**53**	**95**	**80**	**74**	**10**	**68**	**49**	**57**	**58**	**37**	**55**
	**Jiangxi**	**84**	**100**	**98**	**91**	**100**	**82**	**100**	**100**	**96**	**59**	**88**	**63**	**58**	**100**	**41**	**78**	**65**	**72**	**89**	**80**	**77**	**64**	**70**	**53**	**56**	**93**	**77**	**74**	**46**	**67**	**50**	**60**	**60**	**55**	**52**
	**Henan**	**81**	**100**	**94**	**89**	**100**	**95**	**100**	**100**	**95**	**64**	**91**	**47**	**78**	**100**	**50**	**84**	**69**	**67**	**85**	**76**	**81**	**48**	**62**	**51**	**50**	**92**	**69**	**68**	**9**	**68**	**43**	**54**	**50**	**34**	**56**
	**Hubei**	**81**	**100**	**98**	**89**	**100**	**94**	**100**	**100**	**93**	**64**	**91**	**75**	**67**	**100**	**51**	**68**	**71**	**71**	**64**	**68**	**75**	**46**	**59**	**65**	**57**	**96**	**83**	**77**	**20**	**78**	**56**	**74**	**68**	**50**	**56**
	**Hunan**	**76**	**100**	**95**	**86**	**100**	**91**	**100**	**100**	**96**	**64**	**91**	**33**	**62**	**100**	**44**	**79**	**59**	**71**	**88**	**79**	**66**	**68**	**67**	**61**	**54**	**96**	**75**	**73**	**38**	**65**	**64**	**86**	**74**	**60**	**52**
	**Inner Mongolia**	**89**	**100**	**95**	**93**	**100**	**89**	**100**	**100**	**89**	**69**	**90**	**94**	**69**	**100**	**56**	**89**	**80**	**70**	**90**	**80**	**66**	**53**	**59**	**58**	**55**	**97**	**85**	**77**	**56**	**66**	**59**	**78**	**70**	**63**	**57**
**Western China**	Chongqing*	**86**	**100**	**96**	**92**	**100**	**90**	**100**	**100**	**95**	**64**	**90**	**0**	**68**	**100**	**51**	**80**	**31**	**72**	**90**	**80**	**75**	**63**	**69**	**68**	**55**	**95**	**76**	**73**	**38**	**74**	**56**	**71**	**67**	**57**	**55**
	**Guangxi**	**79**	**100**	**97**	**88**	**100**	**92**	**100**	**100**	**94**	**63**	**91**	**0**	**34**	**100**	**45**	**68**	**25**	**68**	**92**	**79**	**79**	**57**	**67**	**63**	**53**	**93**	**80**	**74**	**49**	**70**	**58**	**86**	**68**	**62**	**49**
	**Sichuan**	**85**	**100**	**97**	**92**	**100**	**88**	**100**	**100**	**96**	**62**	**90**	**0**	**66**	**100**	**46**	**79**	**30**	**68**	**87**	**77**	**72**	**57**	**64**	**64**	**49**	**92**	**74**	**70**	**39**	**69**	**53**	**65**	**63**	**55**	**51**
	**Guizhou**	**67**	**96**	**97**	**81**	**100**	**71**	**100**	**100**	**86**	**48**	**81**	**0**	**36**	**100**	**43**	**70**	**25**	**63**	**85**	**73**	**72**	**73**	**73**	**50**	**43**	**86**	**48**	**56**	**58**	**58**	**46**	**49**	**59**	**54**	**39**
	**Yunnan**	**83**	**100**	**98**	**90**	**100**	**92**	**100**	**100**	**90**	**56**	**88**	**0**	**45**	**99**	**43**	**75**	**27**	**68**	**76**	**72**	**55**	**64**	**59**	**59**	**49**	**86**	**57**	**62**	**71**	**66**	**46**	**53**	**55**	**59**	**43**
	**Tibet**	**78**	**100**	**99**	**88**	**55**	**85**	**64**	**64**	**38**	**43**	**56**	**79**	**0**	**100**	**17**	**82**	**25**	**56**	**91**	**71**	**86**	**70**	**78**	**37**	**49**	**42**	**90**	**57**	**90**	**63**	**32**	**35**	**40**	**51**	**30**
	**Shaanxi**	**86**	**100**	**97**	**92**	**100**	**94**	**100**	**100**	**96**	**62**	**91**	**83**	**70**	**100**	**43**	**86**	**74**	**70**	**89**	**79**	**72**	**49**	**59**	**56**	**55**	**95**	**71**	**72**	**33**	**68**	**56**	**70**	**66**	**54**	**53**
	**Gansu**	**82**	**100**	**98**	**90**	**100**	**87**	**100**	**100**	**96**	**52**	**87**	**91**	**68**	**100**	**40**	**86**	**73**	**69**	**86**	**77**	**74**	**64**	**69**	**49**	**49**	**92**	**67**	**67**	**43**	**58**	**45**	**50**	**53**	**49**	**50**
	**Qinghai**	**83**	**100**	**99**	**91**	**95**	**86**	**100**	**97**	**82**	**57**	**85**	**73**	**47**	**100**	**36**	**86**	**64**	**59**	**86**	**71**	**67**	**67**	**67**	**30**	**43**	**88**	**44**	**55**	**53**	**69**	**48**	**56**	**57**	**56**	**39**
	**Ningxia**	**88**	**100**	**98**	**93**	**100**	**95**	**100**	**100**	**96**	**60**	**90**	**83**	**67**	**100**	**49**	**88**	**75**	**70**	**92**	**80**	**80**	**57**	**68**	**29**	**53**	**94**	**77**	**73**	**39**	**73**	**51**	**60**	**61**	**54**	**54**
	**Xinjiang**	**87**	**100**	**96**	**92**	**83**	**94**	**91**	**88**	**90**	**67**	**85**	**0**	**0**	**100**	**48**	**87**	**13**	**63**	**93**	**77**	**83**	**59**	**70**	**37**	**45**	**87**	**66**	**64**	**13**	**71**	**57**	**74**	**69**	**44**	**42**
	Hong Kong**	**95**	**100**	**0**	**31**	**100**	**98**	**100**	**100**	**100**	**75**	**95**	**91**	**82**	**100**	**69**	**86**	**85**	**83**	**87**	**85**	**82**	**78**	**80**	**97**	**78**	**94**	**98**	**90**	**67**	**84**	**61**	**75**	**73**	**71**	**75**
	Macao**	**91**	**100**	**90**	**93**	**100**	**98**	**100**	**100**	**100**	**76**	**95**	**26**	**80**	**100**	**62**	**86**	**65**	**83**	**90**	**86**	**88**	**0**	**9**	**94**	**75**	**97**	**97**	**89**	**59**	**80**	**62**	**76**	**73**	**68**	**78**

*Municipalities directly controlled by central government.

**Special Administrative Region of China; data from Taiwan were not included.

Abbreviations: Cov, coverage; FP, family planning; Hep, hepatitis; HH, household; Incid, incidence; Inj, injury; Mat, maternal; Mod, modern contraception methods; Mort, mortality; NCD, noncommunicable disease; NTD, neglected tropical disease; PM, particulate matter; Poll, pollution; Prev, prevalence; SDG, Sustainable Development Goal; TB, tuberculosis; UHC, universal health coverage; WaSH, water, sanitation, and hygiene

The current situation and recent changes of health-related SDG indicators or their domestic proxies are presented in Table 3 and are described below by thematic topic. Additional insights from qualitative data on the four key topics of child nutrition, MCHRH, TB, and NCDs are summarized in Box 1.

**Table 3:** Current performance of key health SDG indicators in China.

Field	Indicator	Year		Source
Child nutrition		**2002**	**2013**	
	Stunting rate (%)	16.3	8.1	a
	Male	17.1	8.7	a
	Female	15.4	7.4	a
	Urban	7.2	4.2	a
	Rural	23.8	11.3	a
	Wasting rate (%)	2.6	2.0	a
	Male	2.8	2.0	a
	Female	2.3	2.0	a
	Urban	2.1	2.4	a
	Rural	3.0	1.5	a
	Overweight rate (%)	6.5	8.4	a
	Male	7.3	9.4	a
	Female	5.5	7.2	a
	Urban	7.7	8.4	a
	Rural	5.5	8.4	a
Maternal and child health and reproductive health		**2010**	**2016**	a
	MMR (per 100,000 population)	30.0	19.9	b
	Urban	29.7	19.5	b
	Rural	30.1	20.0	b
	U5MR (‰)	16.4	10.2	b
	Urban	7.3	5.2	b
	Rural	20.1	12.4	b
	NMR (‰)	8.3	4.9	b
	Urban	4.1	3.0	b
	Rural	10.0	5.7	b
Infectious diseases	Incidence rate of (per 100,000 population)			
	HIV/AIDS	2.6	4.0	c
	Sex ratio (Male/Female)	3.4	3.6	d
	TB	74.3	61.0	c
	Sex ratio (Male/Female)	1.8	1.8	d
	Malaria	0.6	0.2	c
	Sex ratio (Male/Female)	1.3	1.0	d
	HBV	79.5	68.7	c
	Sex ratio (Male/Female)	1.7	1.7	d
	Brucellosis	2.5	3.4	c
	Dengue	0.0	0.2	c
	Rabies	0.2	0.1	c
	Schistosomiasis	0.3	0.2	c
Noncommunicable diseases and mental health	Premature death rate (per 100,000 population)			
	Cardiovascular disease	320.0	290.8	d
	Male	350.7	332.1	d
	Female	287.1	247.0	d
	Neoplasm	191.4	174.0	d
	Male	243.0	226.3	d
	Female	136.2	118.5	d
	Diabetes	11.3	10.3	d
	Male	10.4	10.2	d
	Female	12.3	10.4	d
	Chronic respiratory disease	83.9	67.0	d
	Male	94.5	79.5	d
	Female	72.6	53.8	d
		**2013**	**2016**	
	Suicide mortality (per 100,000 population)	7.69	7.05	
	Male	8.7	8.1	e
	Female	6.7	6.0	e
	Urban	5.3	4.9	e
	Rural	8.8	8.1	e
Road injury		**2010**	**2015**	
	Road injury mortality (per 100,000 population)			
	Urban total	8.7	13.2	c
	Urban male	12.2	18.8	c
	Urban female	5.0	7.4	c
	Rural total	15.3	19.3	c
	Rural male	22.9	28.6	c
	Rural female	7.4	9.6	c
Environment		**2014**	**2016**	
	PM2.5 annual average (μg/m³)	62	47	f
		**2010**	**2016**	
	Percentage of water quality better than grade III (%)	60.0	67.8	f
	Percentage of rural households with piped sanitation (%)	45.0	60.5	f
Universal health coverage	Contraception rate in married women of childbearing age (%)	89.1	83.0	c
	Prenatal examination rate (%)	94.1	96.6	c
	Hospital delivery rate (%)	97.8	99.8	c
		**2012**	**2015**	
	Antivirus treatment rate (%)	33.3	67.0	g
		**2015**	**2016**	
	TB treatment rate (%)	87.0	87.0	h
		**2008**	**2013**	
	Vaccination coverage (%)			
	Polio	92.4	93.7	i
	Tdap	90.7	92.5	i
	Measles	92.1	97.3	i
	HBV	93.3	93.3	i
	BCG	98.8	98.7	i
		**2010**	**2013**	
	Treatment rate (%)			
	Hypertensive patients over 18	28.7	32.5	j
	Diabetes patients over 18	33.4	35.6	j
	Control rate (%)			
	Hypertensive patients over 18	4.9	9.7	j
	Diabetes patients over 18	34.7	33.0	j
		**2010**	**2015**	
	Number of health professionals (per 1,000 population)	4.4	5.8	c
	Doctors	1.8	2.2	c
	Rural doctors	1.1	1.1	c
	Registered nurses	1.5	2.4	c
		**2010**	**2016**	
	Government health expenditure (%)	28.7	30.0	k
	Social health expenditure (%)	36	41.2	k
	Out-of-pocket health expenditure (%)	35.5	28.8	k
	THE as share of GDP (%)	4.8	6.2	k
		**2013**	**2015**	
	Inpatient reimbursement ratio (%)			
	Urban employee basic medical insurance	55.0	53.9	l
	Urban resident basic medical insurance	46.5	48.2	l
	New rural cooperative medical scheme	39.4	34.6	l
		**2008**	**2013**	
	Proportion of low-income families with catastrophic health expenditure (%)	14.0	16.4	i
	Urban	9.8	8.1	i
	Rural	15.6	19.2	i

The grade III of water quality is defined in a national standard of environmental quality for surface water in China (GB 3838–2002). It is mainly applicable to second class of protected areas for centralized sources of drinking water, wintering farm and migration channel of fish and shrimp, aquaculture and other fishing waters, and swimming areas.

Data source:

a: *Report on Chinese Nutrition and Health Surveillance*.

b: China Maternal and Child Health Surveillance.

c: *China Health and Family Planning Statistics Yearbook*.

d: Global Burden of Diseases Study 2016.

e: China Cause of Death Surveillance Dataset 2016.

f: *Report on the State of the Environment in China*.

g: *China AIDs Response Progress Report 2012–2015*.

h: *WHO Global Tuberculosis Report*.

i: *Analysis Report of National Health Service Survey in China*.

j: *Report on Chronic Disease Risk Factors Surveillance in China*.

k: *China National Health Accounts Report 2016*.

l: *Report on The People's Livelihood Survey in China (2016)*.

Abbreviations: BCG, Bacille Calmette-Guerin; GDP, gross domestic product; HBV, hepatitis B virus; MMR, maternal mortality ratio; NMR, neonatal mortality rate; PM, particulate matter; SDG, Sustainable Development Goal; TB, tuberculosis; Tdap, tetanus–diphtheria–acellular pertussis; THE, total health expenditure; U5MR, under-5 mortality rate; WHO, World Health Organization

**Box 1. Selected qualitative data on child nutrition, MCHRH, TB, and NCDs**

**a. Perceived drivers of child overweight and obesity increase**

Interviews with 18 key informants explored their perceptions of the reasons for increasing overweight and obesity among children in China. Interviewees tended to attribute obesity to individual and societal cognitive and behavioral factors such as parental lack of knowledge about healthy eating, indulgence of children, and changing social norms of a healthy weight.

During the summer or winter vacation, parents do not control the lifestyle of children and indulge them in eating snacks so that children’s weight can sometimes increase up to 5–10 kg.—Technical officer from an international organization

Informants also identified a range of structural influences on children’s diets and levels of physical activity, including parental employment conditions leaving limited time for cooking at home and subsequent reliance on “fast foods,” lack of cooking facilities on schools, lack of controls on dietary provision in and around schools, pressures on children’s time due to prioritization of academic work, and lack of available or affordable sports facilities.

[At home] as parents are busy, it is quite common [for the family] to have meals outside [at a restaurant], where they love to use a big flame and a lot of oil due to the fast speed of cooking, leading to the high intake of fat and energy of children.—Professor in a university, Hubei province

Some schools have set vending machines that sell sodas. Some schools can order take-outs online, where it is impossible to control the nutrition intake. Some small venders are around the school selling roast squid and grilled sausage, and students will eat these when they come by after school.—Official at provincial level

The elementary school … are usually set in the center of residential areas, and they don’t even have a playground or have a very small one … The sports fields are charging high fees to students. The badminton field and swimming pool are charging by 40–50 RMB Yuan per hour.—Official at provincial level

**b. Unmet family planning needs among unmarried women**

Although there are no nationally representative data collected on family planning prevalence among unmarried women, several key informants felt that this group faces unmet needs for contraceptives because they are not provided with free services under the current policy, despite increasing prevalence of premarital sexual activity. In addition, low-income and socially marginalized women in urban areas, such as rural-to-urban migrants, may experience lower financial access and/or entitlement to services.

Contraceptive medicine and devices are free to rural married women. Urban areas and unmarried women are excluded. This issue was brought up during the revision of the Law on Family Planning, but it was still not changed in the amendment.—Former official at the national level

Adolescents are particularly vulnerable to having unmet family planning needs because there is no policy provision for school-based sexuality education.

I think that the increasing of early sex and premarital sex activities is irreversible, but among the department of education, health, and the Youth League, none of them are facing this issue directly. Therefore, there is a blank at both policy and plan level.—Former official at the national level

**c. Multidrug-resistant TB burden and control challenges**

Interviews with a dozen of key informants at the national and provincial levels found that with the increasing number of people screened for multidrug-resistant TB (MDR-TB) and the adoption of new diagnostic methods (GeneXpert), the MDR-TB notified cases in China are increasing. However, there are no accurate data on the number of MDR-TB patients in China.

The MDR-TB screening rate keeps increasing, and it reached the national requirement of 85% in 2016.—Official at the provincial level

One big issue is that we are not clear about the number of MDR-TB patients in China.—Official at the national level

Almost all key informants expressed that the control of MDR-TB faces challenges in case notification, diagnosis, and treatment due to insufficient service coverage and capacity for delivery and difficulty in affording the care. MDR-TB diagnosis and treatment services are not available in some regions. In places where such services are available, the high treatment costs, low actual reimbursement rate (varied based on local health insurance scheme policy), and long treatment duration (around 2 years) reduce patient adherence. Furthermore, low service delivery capacity makes such services largely unstandardized, especially in economically disadvantaged regions.

The adoption of new diagnostic methods, particularly at the primary level is low. The traditional TB sputum smear and culture test capacity is low, and there is a lack of health professionals to do it.—Official at national level

There are huge issues after notifying MDR-TB patients. Even if the costs can be reimbursed as high as 70%, patients still cannot afford the other 30% and refuse to receive the treatment.—Official at provincial level

**d. Progress and challenges for smoking control in China**

One informant at the national level reported that legislation against smoking in public places has been effective in reducing passive smoking in cities where it has been passed. However, many local governments are reluctant to pass similar legislation, let alone implement the policy.

It is a pity that there is no smoking ban legislation in public places at the national level. There are only 18 cities who have done it, covering only 10% of the entire population.—Official at the national level

Legislation is effective; however, it is difficult to do it in China. Now it is NGOs and the general public that are pushing the government to do it. The government attitude toward it is very cautious.—Official at provincial level

Several interviewed informants at the national and provincial level outlined a number of challenges to developing and implementing tobacco control polices, including the power of the tobacco industry (particularly due to its revenue generation), public pressure due to the huge number of smokers, and the limited financial and human resources allocated to smoking control.

To implement smoking control strategies is challenging in China. First, China has a large number of smokers, and it is not easy to change such an addictive behavior. There is also low awareness [about the health harm]. The financial resources allocated to smoking control are limited. Currently, there is only about 30 million RMB coming from central and local government and projects for smoking control use. There are no more than 30 smoking-control professionals in China, and there is only [one person allocating half of their time] working on smoking control in the health education department of CDC.—Official at national level

**Fig 1:**
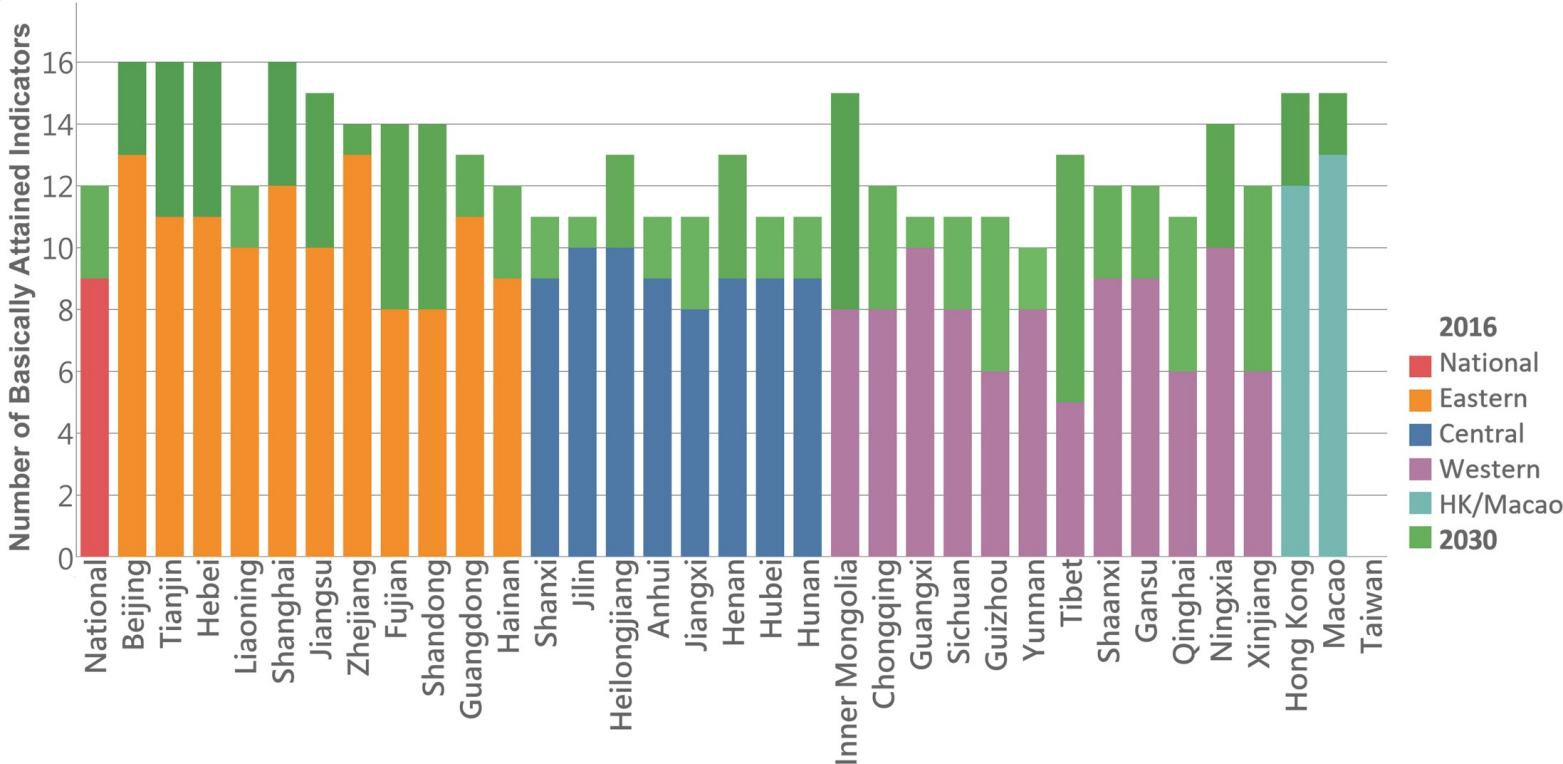
The number of the key health SDG monitoring indicators achieved in China by 2030. SDG, Sustainable Development Goal.

**Fig 2:**
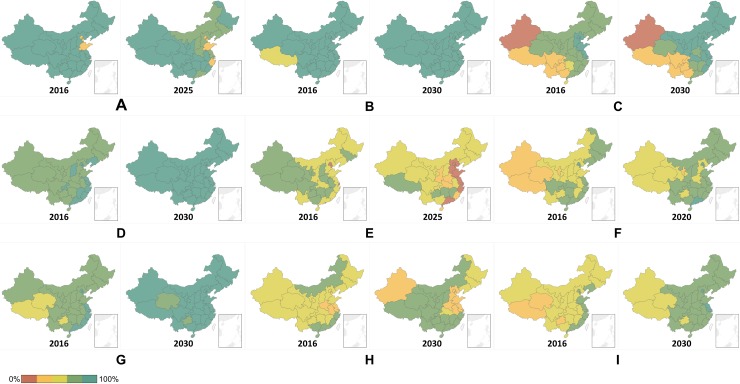
The projected progress of SDG monitoring indicators in China from 2016 to 2030. Calculated based on the GBD 2016 results. Data provided here do not include Taiwan. (A) Child nutrition. It includes three indicators: prevalence of stunting aged under 5 years (SDG 2.2.1), prevalence of wasting aged under 5 years (SDG 2.2.2a), and prevalence of overweight aged 2–4 years (SDG 2.2.2b). (B) Maternal and child health and reproductive health. It includes six indicators: maternal mortality (SDG 3.1.1), skilled birth attendance (SDG 3.1.2), under-5 mortality (SDG 3.2.1), neonatal mortality (SDG 3.2.2), vaccine coverage (SDG 3.b.1), and family planning need met with modern contraception methods (SDG 3.7.1). (C) Infectious diseases. It includes five indicators: HIV incidence (SDG 3.3.1), TB incidence (SDG 3.3.2), malaria incidence (SDG 3.3.3), hepatitis B incidence (SDG 3.3.4), and prevalence of neglected tropic diseases (SDG 3.3.5). (D) NCDs and mental health. It includes two indicators: morality due to cardiovascular diseases, cancer, diabetes, and chronic respiratory diseases at age 30–70 (SDG 3.4.1) and suicide mortality (SDG 3.4.2). (E) NCD risk factors. It includes two indicators: smoking prevalence (SDG 3.a.1) and alcohol use (SDG 3.5.2). (F) Road injury. It includes one indicator: road injury mortality (SDG 3.6.1). (G) Environmental health outcomes. It includes three indicators: air pollution mortality (SDG 3.9.1), WaSH mortality (SDG 3.9.2), and poisoning mortality (SDG 3.9.3) were related to health outcomes. (H) Environmental exposure risk factors. It includes five indicators: mean PM2.5 level (SDG 11.6.2), household air pollution (SDG 7.1.2), unsafe or unimproved water sources (SDG 6.1.1), sanitation (SDG 6.2.1a), and hygiene (SDG 6.2.1b). (I) UHC index. It includes one indicator: universal health coverage index (SDG 3.8.1). GBD, Global Burden of Disease; NCD, noncommunicable disease; PM, particulate matter; SDG, Sustainable Development Goal; UHC, universal health coverage; WaSH, water, sanitation, and hygiene.

Our analysis estimated that China may achieve 12 of the 28 selected health-related SDG indicators in 2030. The total number of indicators achieved varied geographically among provinces, and two-thirds of the provinces are estimated not to be able to achieve half of the 28 indicators, though the geographic gap was narrowed (Fig 1). Eastern coastal provinces are predicted to perform better than central and western provinces, especially for Beijing, Tianjin, Shanghai, and Hebei, which could achieve 16 indicators. However, western provinces progressed faster than eastern and central provinces compared with baseline status, especially for Tibet, which could achieve eight more indicators compared with its baseline in 2015, ranking first among all the provinces. Fig 2 presents the projected progress of the attainment index of the indicators by province from 2015 to the target year, and details are described by thematic topic below.

### Current performance and future attainment of health-related SDG indicators by thematic topic

#### Child nutrition

Child undernutrition greatly improved over the past decades, whereas overweight and obesity emerged as a new public health challenge in China. Among children aged 6 years and younger, the stunting rate reduced from 16.3% to 8.1%, and wasting reduced from 2.6% to 2.0% from 2002 to 2013. However, overweight rates increased from 6.5% to 8.4% from 2002 to 2013. The perceived drivers of the increase were explored in our interviews (Box 1) [18].

Sex and regional disparities existed in child nutrition. In 2013, the child stunting rate and overweight rate were 1.2 and 1.3 times higher among boys, whereas there were no sex disparities in wasting. Child stunting and wasting rates were 2.7 and 1.6 times higher, respectively, in rural than in urban areas. There was no urban–rural difference in child overweight rate. Eastern provinces had lower rates in child stunting and wasting but higher rates in child overweight than central and western provinces.

Based on the projections of the three included indicators, it is estimated that the performance of child nutrition in China will worsen by 2025 if current trajectories are not addressed (Fig 2A). This is due largely to a rapid increase in childhood overweight and obesity seen almost everywhere in China. The attainment index is projected to drop to 80.5 in 2025 from 91.1 in 2016, with an annual change rate of −1.1. Such a sharp decrease is especially obvious among eastern provinces and a few western provinces. Shandong, Tianjin, and Shanghai ranked as the top three in terms of the projected rapid decrease. In terms of sex disparities, the child overweight rate is projected to be 1.4 times higher among boys in 2025. China will fail to achieve the zero-increase target for child overweight rate if actions are not taken. Though China is estimated to perform well in reducing stunting and wasting, sex disparities remain: the stunting and wasting rate is estimated to be 1.2 and 1.4 times higher, respectively, among boys in 2025.

#### MCHRH

China has achieved remarkable progress in MCHRH over the past decades. The maternal mortality ratio (MMR), under-5 mortality rate (U5MR), and neonatal mortality rates (NMRs) have decreased to very low levels, reaching 19.9/100,000, 10.2‰, and 4.9‰ in 2016, respectively [3]. The vaccine coverage among children under 5 years old was over 90% in 2013 [19]. However, other aspects of reproductive health show a mixed picture. For example, the contraception rate among married women was 83.0% in 2016, whereas the availability of contraceptive services among unmarried women was low, based on published studies [19,26] and the results from our interviews with key stakeholders (Box 1).

Despite the overall excellent performance, regional disparities exist among urban and rural areas and geographic areas. In 2016—except for MMR, which had almost no urban and rural differences—the NMR and U5MR were 1.9 and 2.4 times higher in rural than urban areas, and the MMR, NMR, and U5MR were 2.0, 2.7, and 3.4 times higher in western provinces compared with eastern provinces.

Based on the projections of the six included indicators, it is estimated that the performance of MCHRH in China will continue to improve toward 2030 based on the historical progress (Fig 2B). The attainment index is projected to increase to 95.3 in 2030 from 91.8 in 2016, with an annual change rate of 1.0. Regional disparities are estimated to narrow: the attainment index of Tibet is projected to increase fastest and reach 87.0 in 2030.

China had already achieved the SDG 2030 targets set for MMR, U5MR, and NMR, and it had a high institutional-based infant delivery rate and almost achieved universal vaccine coverage in 2016. However, the attainment index of family planning needs met with modern contraception methods, which is projected to reach 76.6 in 2030, is not as high, largely due to the unmet needs among unmarried reproductive women.

#### Infectious diseases

The notified cases of TB, malaria, and hepatitis B decreased in the past decade, whereas the new notified HIV/AIDS cases increased steadily, though at a low level. In 2016, the hepatitis B notification case rate ranked first among all infectious diseases, reaching 68.7/100,000, followed by TB at 61.0/100,000, HIV/AIDS at 4.0/100,000, and malaria at 0.2/100,000 [3]. However, we found through our interviews that the notified new cases of MDR-TB have been increasing over the past years, and its control faces great challenges (Box 1). Only the incidence of four neglected tropical diseases were officially reported (brucellosis, dengue, rabies, and schistosomiasis), totaling 3.85/100,000 in 2016.

Sex disparities were significant for infectious diseases indicators. The incidence of HIV/AIDS, TB, and hepatitis B were higher among males, which were 3.5, 1.8, and 1.7 times higher than females, respectively.

The performance of infectious diseases in China will continue to improve toward 2030, judging from projection results of the five monitoring indicators based on past trends (Fig 2C). The attainment index is projected to increase to 73.8 in 2030 from 63.0 in 2016, with an annual change rate of 0.7. The improvement is especially obvious among eastern and a few central provinces. Guangxi, Sichuan, and Yunnan ranked as the top three in terms of the projected increase. Eastern and central provinces are projected to perform better than western provinces by 2030 in terms of achieving the targets, especially Shandong, Hong Kong, Tianjin, and Jiangsu, which are projected to score above 88.0 in 2030.

China will also achieve the ending malaria target by 2030, as all provinces scored 100.0 in the attainment index in 2030. However, the challenges of ending HIV/AIDs and TB will be tremendous: the attainment index is projected to be 53.8 and 75.8, respectively, in 2030 and is lowest among western provinces. It is promising for China to end neglected tropical diseases by 2030, as the attainment index is projected to reach 87.1, though a few southern provinces had lower scores. Combating hepatitis will be difficult: the attainment index of hepatitis B incidence is projected to increase to 61.8 in 2030, demonstrating the challenges China faces in controlling hepatitis.

The HIV/AIDS and TB targets are projected to be achieved faster for women than men. The incidence of HIV/AIDS and TB are projected to decrease by 29.8% and 33.3%, respectively, among women from 2016 to 2030, whereas they only decrease by 7.9% and 14.7%, respectively, among men. Sex disparities in the incidence of hepatitis B virus (HBV) are projected to reduce, with a similar percentage decrease at around 55%, though the projected male incidence is still 1.8 times higher than female incidence.

#### NCDs and mental health

NCDs remain the leading cause of death in China, though the past decades saw substantial decreases in age-standardized mortality [3]. The age-standardized mortality of the four major NCDs was 542.1/100,000 in 2016, with cardiovascular diseases ranking first. Suicide mortality rate has decreased over the past decade, reaching 4.9/100,000 among urban areas and 8.1/100,000 among rural areas in 2016 [20]. The harmful alcohol consumption rate among drinkers increased from 3.3% in 2007 to 8.8% in 2013 [21]. The smoking rate (≥15 years old) experienced a slow decrease over the past 2 decades, reaching 27.7% in 2015 [27]. Challenges related to smoking control were examined in our interviews (Box 1).

Sex disparities were substantial among NCD indicators. Male mortality rates were higher than females’, except for diabetes: the baseline numbers of males were 1.5, 1.4, 5.9, and 19.3 times higher than that of females in NCD mortality, suicide rate, harmful alcohol consumption rate, and smoking rate, respectively.

We have done the projections using two mortality indicators related to premature deaths due to NCDs and suicide. These two indicators will greatly improve by 2030 based on China’s historical progress (Fig 2D). The attainment index is projected to increase to 100.0 in 2030 from 79.7 in 2016, with an annual change rate of 1.6. The attainment index of all provinces is projected to reach above 90.0 in 2030. However, provinces are projected to perform better in suicide mortality than premature NCD morality. Although the attainment index of all provinces is projected to reach above 95.0 in suicide mortality, three provinces, Guangxi, Yunnan, and Hubei, are projected to score under 90.0 in NCD premature morality. Sex disparities are projected to exist in both NCD premature death and suicide. The premature probability of dying from the four NCDs is projected to drop by 21.5% among males compared with 38.9% among females from 2016 to 2030. Projection results show the suicide rate will decrease faster among females at 51.6% than males at 21.5%.

Based on the projections of the two indicators of NCD risk factors, it is estimated that the performance of NCD risk factors in China will continue to worsen by 2025 if the current trajectories continue (Fig 2E). The attainment index is projected to drop to 38.8 in 2025 from 60.5 in 2016, with an annual change rate of −1.3. Such sharp decreases are especially obvious among eastern provinces. Beijing, Tianjin, and Shanghai rank in the top three in terms of the projected decreasing speed.

The worsening of performance in NCD risk factors is largely attributed to the significant increase of alcohol use, though the smoking prevalence is projected to decrease. In terms of sex disparities, the male smoking prevalence (≥15 years) is projected to be 12.3 times higher than females in 2025. In addition, the male smoking prevalence is projected to decrease slower than females. Smoking in males is projected to decrease by 8.4% compared with a decrease in females by 13.1% from 2016 to 2025. The male daily alcohol consumption in the past 12 months (≥15 years) is projected to be 14.0 times higher than for females in 2025, and males are projected to experience an increase of 17.0% compared with 15.8% among females.

### Road injuries

Road injury mortality in China has had a steady decrease since 2013, after years of increase, dropping to 19.3/100,000 in rural areas and 13.2/100,000 in urban areas in 2015 [3]. The road injury mortality rate among males was 2.7 times higher than females in urban areas and 2.9 times higher in rural areas [3].

China’s performance in road injury mortality is projected to improve based on its historical progress (Fig 2F). The attainment index is projected to increase to 70.0 in 2020 from 63.6 in 2016, with an annual change rate of 1.6. Qinghai, Xinjiang, and Henan ranked as the top three in terms of the speed of improvement. Despite the rapid progress among a few western provinces, eastern and central provinces are projected to perform better than western provinces by 2020. Ningxia, Qinghai, Tibet, and Xinjiang are projected to score below 50.0 in 2020, demonstrating large improvements in road safety. In terms of sex differences, the road mortality is projected to decrease by 8.3% among women but only 4.8% among men. China will seemingly fail to achieve the 50% reductions in road injury mortality by 2020.

### Environmental health

For air pollution, the overall trend has been decreasing, though it remains high especially in urban areas. Nationwide, the PM2.5 dropped from an annual average of 64 μg/m^3^ in 2014 to 47 μg/m^3^ in 2016. The Beijing–Tianjin–Hebei, Yangtze River Delta, and Pearl River Delta regions were 39.6%, 34.3%, and 27.7% lower than those of 2013, respectively [28]. However, the average levels of PM2.5 were 58 μg/m^3^ and 39 μg/m^3^ in Beijing and Shanghai, respectively, in 2017 [28]. The surface water quality continued to improve, with the percentage of water quality better than grade III increasing to 67.8% in 2016 [22]. The percentage of rural households with piped sanitation increased steadily from 45.0% in 2010 to 60.5% in 2016 [3].

Based on the projection results of the three indicators related to environmental health mortality, China’s performance will continue to improve toward 2030 (Fig 2G). The attainment index is projected to increase to 89.2 in 2030 from 75.5 in 2016, with an annual change rate of 0.9. The improvement is especially fast among some western provinces. Tibet, Guizhou, and Yunnan ranked as the top three in terms of the projected increasing speed. Because of the estimated rapid progress among western provinces, regional disparity gaps are projected to be reduced, and the majority of provinces will score above 80.0 in the attainment index by 2030.

Looking separately into the projection results of the three monitoring indicators, China is projected to perform best in WaSH mortality by 2030, followed by poisoning mortality and air pollution mortality. The attainment index of WaSH and poisoning mortality is projected to increase to 99.3 and 89.6 in 2030, whereas the number is 79.7 for air pollution mortality. Air pollution may continue to be a serious problem in some Chinese provinces. The attainment index of air pollution mortality is projected to be even lower among a few western provinces, especially for Qinghai, Xinjiang, and Guizhou, with the projected score under 70.0 in 2030.

China’s performance in environmental exposure risk factors is projected to improve, though slowly, toward 2030, based on the past trends of the five indicators (Fig 2H). The attainment index is projected to increase to 60.2 in 2030 from 54.9 in 2016, with the annual change rate of 0.4. Though the overall performance is projected to improve, the performance of some provinces is projected to worsen. Among provinces that are projected to have better performance, Yunnan, Guizhou, and Gansu score highest. However, there are a total of nine provinces that are projected to have poorer performance in environmental exposure risk factor indicators, and most of them are economically developed and resourced provinces. The deterioration speed is projected to be fastest in Beijing if current trajectories are not addressed.

Looking separately into the projection results of the five monitoring indicators, the performance of household air pollution, sanitation, hygiene, and water is projected to improve, except for that of the PM2.5 level. The attainment index of household air pollution, sanitation, hygiene, and water is projected to increase to 91.0, 88.0, 78.6, and 67.8 in 2030, whereas the attainment index of PM2.5 is projected to decrease to 25.5. Such decreases are particularly obvious among provinces in the north and eastern parts of China and the western province Xinjiang.

### UHC

There were two monitoring indicators for UHC used globally: coverage of essential health services (SDG 3.8.1) and large household expenditures on health (SDG 3.8.2), though the GBD Study 2016 only included UHC index to present the essential health services coverage because of data availability [23]. China had made great progress in expanding health services coverage for maternal, neonatal, and child health; infectious diseases; and NCDs, though the treatment and control rate for hypertension and diabetes remained low. For example, the hypertension and diabetes control rates among people over 18 were only 9.7% and 33.0%, respectively, in 2013. Service capacity has improved, and the number of total health professionals, doctors, and nurses kept increasing, though urban–rural disparities remain (Table 3). The total health expenditure (THE) has increased since 2000, and the out-of-pocket payment (OOP) ratio decreased to 28.8% in 2016 [3]. China has almost achieved universal coverage for basic health insurance. The percentage of low-income households (lowest quintile) with over 40% of household income spent on health increased from 14.0% in 2008 to 16.4% in 2013 [19]. Residents in rural areas suffered greater financial burden from health expenditures, with the percentage 2.4 times higher than that of urban residents in 2013.

Based on the projection results of the UHC index, a measure of the essential services coverage alone, China’s performance in UHC is projected to improve toward 2030 based on the past trends of the five indicators (Fig 2I). The attainment index is projected to increase to 69.2 in 2030 from 54.4 in 2016, with an annual change rate of 1.1. The improvement is especially obvious among western and central provinces. Guizhou, Xinjiang, Gansu, and Tibet ranked top four in terms of the projected increasing speed. Nevertheless, eastern and central provinces are projected to perform better than western provinces by 2030, especially Macao, Hong Kong, Beijing, Shanghai, and Jiangsu, which are projected to score over 80.0. China will fail to achieve UHC by 2030 based on the UHC index results if following the historical trajectories. The challenges will become even more formidable when taking the financial protection coverage into consideration.

## Discussion

### Summary of key findings and challenges

China has already achieved several health-related SDG targets proposed by the UN, including the NMR, U5MR, and MMR. In the MDG era, the Chinese government—with support from the international community—made significant efforts to tackle both communicable diseases and maternal and child health. However, our study found that there are six particular challenges in the SDG era: control of hepatitis and MDR-TB, curbing NCDs, achieving UHC, tackling regional inequities, and population aging.

First, China does not have a comprehensive hepatitis strategy, though it supports selected components (HBV vaccination for children, preventing transmission from mother to baby, and some hospital-based safety measures) [29]. Rates of case notification and effective treatment of hepatitis are low because of several factors, such as poor diagnosis, high healthcare costs, and stigma [30–33]. A significant proportion of people infected with HBV and hepatitis C virus (HCV) will develop liver cirrhosis, cancer, or other serious liver diseases [34,35].

Second, China has the second largest number of patients with MDR-TB—the most recent survey, conducted in 2010, estimated that there are about 339,000 people with MDR-TB [36]. China did not start to implement an effective strategy for addressing MDR-TB until the Global Fund (GF) supported China to undertake MDR-TB case finding and treatment in a small number of Chinese provinces in 2003 [37]. The GF-funded project has ended because China no longer qualifies for GF funding. More recently, with support from the Bill & Melinda Gates Foundation, China started to develop and implement an MDR-TB control program slowly in a limited number of provinces [38]. Nevertheless, the case notification rate is still very low. In 2016, only 2,016 patients with MDR-TB were detected, of which only 68% have been put on treatment [36]. Apparently, China is not on course to achieve the target of ending TB by 2030 set out by WHO.

Third, although our study suggests that China may be able to reach the SDG 3 target on NCDs (i.e., reduce almost one-third of premature deaths due to NCDs by 2030), it still faces significant challenges in controlling NCDs. First, premature mortality due to NCDs among men is not predicted to decline significantly by 2030, owing largely to continuing risk factors, such as smoking, drinking, and physical inactivity. Second, there is no sign that the rate of overweight and obesity will decline in China in the near future. Third, there are insufficient prevention and health promotion efforts to address risk factors associated with NCDs, such as reducing air pollution, public smoking bans, reduction in harmful alcohol consumption, control of hypertension, and reducing salt intake [39]. Fourth, China has a major burden of mental illness. In 2016, there were an estimated 56 million people with depression, 44 million with anxiety, and 16 million with attention deficit/hyperactivity disorder based on the GBD Study 2016 results. Despite this huge disease burden, the health system response to NCDs and mental health issues in China has been inadequate. Key informants argued that NCD spending was too heavily weighted toward treatment rather than risk reduction. For mental health problems, the insufficient budget is compounded by stigma, low rates of case notification, diagnosis and treatment (especially for nonsevere mental disorders), and low quality of mental health services. Furthermore, the weak capacity of the primary care system is a barrier to effective control of NCDs, including mental illness.

Fourth, China faces many obstacles to expanding access to health services while also providing financial protection. The Chinese government has increased its investment in health significantly in its 12th five-year plan. During 2011–2016, the average annual increase in the rate of government expenditure on health was 17.3% [3], much faster than the economic growth rate. As a result, access to essential health services has improved in recent years. OOP, as a percentage of total health expenditure, has declined significantly in recent years. Nevertheless, in 2016, THE accounted for 6.2% of GDP in China, of which 28.8% was still paid for by OOP costs [3]. Healthcare costs are rising rapidly: THE increased from USD 55 billion in 2000 to USD 658 billion in 2015 [3]. However, about 70% of this was spent on treatments, whereas only slightly over 5% was invested on public health programs in 2015 [3]. Our study found that effective reimbursement rates by health insurance schemes were between 55% and 35%: the highest rate was offered by the urban employee basic health insurance (UEBHI), and the lowest rate was offered by the new rural cooperative medical scheme (NRCMS). The reimbursement for inpatient service expenses had declined for the UEBHI and NRCMS from 2013 to 2015. Consequently, the financial burden of healthcare placed on service users, particularly the poor in rural areas, remains very high, and the percentage of households with catastrophic health expenditure in the rural areas has increased for the people at the lowest income quintile [16].

Fifth, a major challenge China faces is huge regional health disparities: on most indicators, the eastern provinces have much better health status and healthcare than the central and western provinces (except for child overweight, NCD risk factor indicators, and environmental exposures indicators). Our study estimates that two-thirds of provinces will not have achieved half of 28 health-related SDG indicators by 2030.

A final but grand challenge that China urgently needs to tackle in the upcoming decades is the rapidly aging population, which will place additional demands on the health system. China has the largest aging population in the world. In 2017, there were over 228 million people aged over 60 years in China, and the number is projected to double by 2050 [40]. The number of those aged 80 years and above is estimated to quadruple over the next 30 years, increasing from 22.6 million in 2013 to 90.4 million in 2050 [41]. The rural–urban migration due to urbanization further contributes to rapid aging in rural areas [42]. These dramatic changes in population age structure will lead to a rising burden of age-related diseases, geriatric syndromes, and caregiving, which has profound implications for China’s healthcare system, which is already facing enormous challenges, such as insufficient allocation of resources and inequalities in availability and accessibility to healthcare services [43,44]. As an emerging policy and public health priority in China, population aging calls for aligning the health system with the needs of the increasing aging population to provide affordable and high-quality health and long-term care services to achieve healthy aging and UHC.

### Comparisons with other relevant studies

The GBD SDG collaborators team is monitoring global progress toward the health-related SDG indicators and has published results based on findings of the GBD 2015 [24], 2016 [23], and 2017 studies [10]. Over the past 3 years, the team has updated the indicators and refined GBD data estimates and its scaling and forecasting methodology. Their recently published study provided subnational and sex disparities analysis [10]. Through using GBD Study 2016 data and a similar projection model, we developed a new scaling methodology to set the worst value observed in 1990–2015 as zero after teasing out outliers and the SDG targets value as 100. The scaled scores imply how far one indicator is from the SDG target in a specific year.

The findings related to SDG target attainment are consistent for most of the indicators between this study and the recent study published by the GBD 2017 SDG collaborators (SDG GBD Study 2017) [10]. Similar to the global situation, it is almost impossible for China to achieve the targets on child overweight, road injuries mortality, and HIV/AIDS and TB incidence. We also found similar results on regional disparities. Both the SDG GBD Study 2017 and the current study found that there are considerable subnational variance in terms of the health-related SDG indicators in China.

Findings related to sex disparities are similar in pattern, though there are differences in the male-to-female ratio of several indicators. Similar to the global situation, men are at higher risk of excess mortality than women and have higher incidence rates for TB, HIV/AIDS, and hepatitis B and higher rates of premature mortality from NCDs, suicide, and road injuries in China. Men also have significantly higher rates of some key NCD risk factors, such as smoking and daily alcohol consumption. This disparity also applies to our future projections. Despite the similar pattern of sex disparities, the male-to-female ratio of several indicators such as smoking prevalence, daily alcohol consumption, and suicide rate have specific features in China. The smoking prevalence and alcohol consumption are around 19 times and 6 times higher, respectively, for the Chinese men than women. In contrast, the male suicide rate only started to exceed females slightly in recent years, whereas in high-income countries, the ratio is about 3 times higher among men [45]. Such extreme and divergent differences in male-to-female ratios call for a more nuanced analysis and customized interventions specific to the context of China to target both sexes and address underlying gender relations.

The findings on sex disparities add to existing global evidence that inequitable gender relations, which privilege men, are nonetheless often harmful to men’s health in many contexts [46,47,48]. These may include social norms of masculinity that encourage high levels of smoking and drinking and potentially discourage help seeking for mental ill-health, as well as greater occupational exposure to physical and chemical hazards. Although higher overweight rates among boys may be in line with well-documented son preference in China, in which more resources will be given to boys than girls [49], as favoring boys in food allocation may contribute toward ill-health in a context of plenty, it is unclear why boys fare worse than girls in terms of stunting and wasting. One possible explanation is that boys have higher energy metabolism than girls as they reach puberty. If boys, especially in rural areas, have poor nutrition during puberty, it is more likely for them to suffer stunting and wasting than girls. Nevertheless, the reasons for this counterintuitive finding require further investigation, as well as more nuanced consideration of intersections with geography, wealth indicators, and birth order, because recent studies have suggested that girls in non-one-child households in rural China face nutritional disadvantage [50].

### Strengths and limitations of the study

To the best of our knowledge, this is the first study that takes an evidence-based approach to systematically analyze China’s progress toward the health-related SDGs and highlight the specific challenges by health topic. The research team used both the estimates from the GBD Study 2016 and domestic public data sources for quantitative analysis and conducted key informant interviews and extensive literature and policy reviews to draw a comprehensive health picture of China. One outstanding feature of the study is that the analysis is stratified by sex and region/province whenever possible to illuminate disparities, complementing the SDG GBD Study 2017 that touches on subnational analysis for several countries including China, but does not present results by province and sex within China. The presentation of goal attainment in the form of a percentage is novel—it conveys a direct message to policy makers in an accessible way. The methodology used in this paper has the potential to be adapted into a longitudinal tracking system to measure China’s attainment of the health-related SDGs. Furthermore, the results of this study can serve as a strong evidence base for the Chinese government and the international community to (1) understand the current situation, (2) recognize challenges and gaps toward achieving the health-related SDGs in China, and (3) help with effective health policy making.

Nonetheless, the study has several limitations related to data availability and quality. We cannot obtain valid data to present a comprehensive picture for several important indicators, such as those related to the unmet need for reproductive health services among unmarried women and MDR-TB epidemiology. Domestic data for some indicators is defined differently from the official SDG indicators, such as rates of child stunting, wasting and overweight, and infectious diseases incidence, making international comparisons challenging. The quality of both domestic data sources and the GBD 2016 estimates has room for improvement. The limitations of the GBD 2016 estimate are largely due to data gaps, methodological challenges, and/or computational constraints [51–55].

Although the current study results are not updated to GBD Study 2017 estimates because of data availability, we have carefully studied the GBD 2017 methodology and compared the changes in the selected 28 health-related SDG indicators. Methodologically, GBD 2017 added new data input sources and used more refined and sophisticated methods for calibration, and they have modeled covariates and risk factors into the projections to boost data quality. Therefore, the estimates of health-related SDG indicators of GBD 2017 are generally more reliable than those of GBD 2016. In terms of the China-specific estimates used in this study, our comparison shows that the estimates of health-related SDG indicators of the GBD Study 2017 at the national level are consistent with those of the GBD Study 2016, except for hepatitis B incidence, suicide mortality, and mean PM2.5 level, in which we found significant changes in either data values or trends. For example, the estimates of PM2.5 in GBD 2017 are now more aligned with the results from the national surveys and surveillances, whereas GBD 2016 estimates tend to overestimate, although the magnitude of change is not large. Yet it remains unclear to us which study provides better estimates in terms of suicide mortality and hepatitis, which may need further investigation.

The exclusion of nine of the GBD indicators from our analysis narrows the assessment of China’s performance on SDGs by focusing on classic and directly health-related indicators. The decision was, however, made by our best judgement based mainly on data availability and quality and potential impact on policy changes. We believe that the excluded nine indicators are an important part of the health-related SDGs and cannot be ignored for a comprehensive performance assessment. Future studies could consider the inclusion of more indicators into the analysis (GBD 2017 included four more new indicators), especially when robust data from domestic sources become available to cross-check the data quality from international and national sources. We therefore call for better national surveillance, or undertaking of national surveys, on these indicators—for example, those related to violence against women and children and occupational risks.

The projection methodology, though carefully selected through comprehensive comparisons, can only reflect future changes based on historical trajectories. First, it fails to capture other possible changes such as a stronger political commitment and new and effective technological advances and interventions. Such changes will impact the results; however, it is impossible for us to predict when and how these would happen, and so we could not build them into the model. Second, the methodology does not consider the possibility of a ceiling effect. For example, without more effective interventions and new investments, it might be extremely difficult to reduce the NCD mortality and prevalence rates after they have reached a low level. In addition, we did not build in demographic, socioeconomic, and other related factors such as population change, life expectancy, GDP, or educational attainment into the model, thus failing to capture the impact of these factors on the burden of disease. However, including such variables into the projection model would require further projection of these variables, involving many assumptions, which may introduce extra biases and uncertainties and undermine the validity of the projection results.

We scaled the indicators in a way that captures how far each indicator is from the SDG targets. However, given that some of the health-related SDGs do not have defined targets, we refer to the international targets issued by WHO if there are no undefined targets and make arbitrary though reasonable targets for those with neither SDG nor WHO targets. In addition, as some of the SDG indicators have relative targets, such as a one-third reduction in premature mortality from NCDs (SDG 3.4), the scaled scores can only reflect the relative progress for these indicators; they mask the performance of absolute values. For example, both Beijing and Chongqing are projected to score 100 in 2030, implying that they can reduce the premature mortality from NCDs by one-third, though the absolute rates are projected to be 153.1/100,000 and 256.8/100,000 in Beijing and Chongqing, respectively, in 2030.

Finally, the specific SDG health-related indicators may also obscure certain challenges and dimensions of health. For example, women typically experience higher rates of NCD morbidity (but not premature mortality) than men [56], but this disparity is not captured by these indicators because morbidity data are absent. Similarly, suicide mortality is not a good overall proxy for mental health, with sex disparities varying according to different mental health conditions globally. Consequently, the indicators under the thematic topics may not comprehensively reflect the health performance in the domain.

### Policy implications

China needs to take concerted actions to overcome challenges and achieve the health-related SDGs. In those areas in which China has already achieved the SDG targets—for instance, in maternal and child health and nutrition—the government should synthesize the experiences and best practices that can be applied to the provinces that need to catch up. These experiences and best practices could also be disseminated to other low- and middle-income countries that are developing their national strategies in advancing the health-related SDGs via South–South cooperation and the Belt and Road Initiative.

The government needs to examine the root causes of the health problems in areas in which China has formidable challenges in achieving the health-related SDGs by 2030 and develop and implement a feasible action plan under the current socioeconomic context of China. Then, the plan should prioritize key policy interventions for the target population and mobilize adequate resources to tackle these health challenges. Our study has identified key areas for action and cost-effective interventions in the context of China based on findings in the *Disease Control Priorities*, *3rd edition*, as shown in Box 2. Given the limited available health resources, it is critically important to identify which populations should be prioritized for which interventions and develop appropriate approaches to reach these targeted populations. For example, reproductive health interventions may be targeted to rural-to-urban migrant women and adolescents and students from universities and middle and high schools. To better control infectious diseases (e.g., TB and hepatitis), poor and other vulnerable groups should be prioritized. In addition, it is important to address common problems by tackling cross-cutting issues in advancing the health-related SDGs. Key strategies include (1) enhancing the enforcement of the health-related laws and regulations, (2) establishing an effective organizational structure of government agencies and defining their responsibilities and functions clearly for implementing health-related SDG targets under the auspices of the State Council, and (3) strengthening the development of human resources for health, especially for the least developed regions.

**Box 2. Key areas for policy change and implementation**

**a. Tackling child overweight and obesity**

International experience shows that increasing taxes on sugar-sweetened drinks, subsidizing healthy foods/diets, implementing school-based nutrition interventions, and regulating food and drink sale markets would have positive impacts on the reduction of overweight and obesity. Therefore, appropriate strategies, policies, laws, and regulations should be developed and implemented to address this rising problem.

**b. Improving breastfeeding**

Many strategies could be used to increase breastfeeding in China: establishing more baby-friendly hospitals and clinics [57]; expanding health education programs, including peer education/support [58]; targeting not only women of reproductive age but also the public as a whole; and setting up more places friendly for breastfeeding at working units and communities.

**c. Enhancing access to reproductive health services including strengthening sexual education**

Induced abortion has increasingly become a very serious problem, particularly among unmarried women and teenage girls in China. The government needs to improve access to reproductive healthcare, particularly for vulnerable groups, and develop more health education programs via social media, public adverts, or school-based education platforms [59,60,61,62]. Contraceptive services and products should be made more easily available and covered by either the government budget or health insurance to all those who need it (not just married women).

**d. Strengthening care and control of key infectious diseases**

China has developed and implemented good policies and taken appropriate actions on HIV/AIDS over the past decades. Nevertheless, China should also develop more proactive policies and actions on tackling TB/MDR-TB and hepatitis B and C—the top three infectious diseases in terms of burden of disease and number of patients. More favorable policies should be developed by health insurance schemes in China to support these patients to seek affordable care, including new diagnostics and drugs, as required [63,64,65]. The government budget earmarked for control of these diseases should be increased, and better used, and funding for public health intervention programs should be allocated toward these priorities.

**e. Developing and implementing effective preventive interventions for NCDs**

Community-based interventions, particularly targeting key risk factors of NCDs, must be developed and implemented in order to prevent more people from developing NCDs. A set of concerted interventions, including the enforcement of relevant health legislation (e.g., implementing the Framework Convention on Tobacco Control); increasing tobacco and alcohol taxes [66,67]; strengthening the control on the packaging and advertising of tobacco products [68,69,70]; ban on public smoking [71,72]; adopting social media to promote healthy lifestyles including reducing the intake of salt, cooking oil, and sugar; increasing physical exercise; and improving air quality, must be developed under the auspices of the national health commission, in collaboration with other relevant ministries, and with the support of local governments at different levels [73].

**f. Developing adequate systems to address the rise in mental health challenges**

China has a significant number of people who require mental health care, especially people with depression and anxiety. However, it has not yet established a system within the health sector to address the needs of, and demands for, mental health care. Prioritized actions might be the development of school-based life skills training for young people [74,75,76,77], and community-based mental care including treatment of depression and other common mental illnesses [76].

**g. Adapting effective interventions to reduce road injuries**

Safety belts [78], safety seats for children [79], and helmets [80] are very cost-effective ways to reduce road injuries. Laws, regulations, and public education should be enforced and implemented at the same time to increase the awareness of the public.

The improvement of the Chinese people’s health status lies not only in health sector development but also in the overall sustainable development of socioeconomic and other sectors. China needs to imbed health into all the national and provincial policies, increase health investments, and improve financing mechanisms to provide better financial protection for those seeking essential healthcare.

## Conclusions

The patterns of mortality and health risk factors shown by our study reflect a rapid health transition in China away from primarily infectious disease burden and toward NCDs and risk factors associated with industrialized societies, although challenges in tackling hepatitis and TB/MDR-TB remain for specific segments of the Chinese population. Addressing these issues will require a paradigm shift that reorients the Chinese public health system toward health promotion and prevention efforts to reduce key risk factors and to strengthen the detection and management of NCDs, particularly in their early stages [81]. There is also a clear need to focus on policies, systems, and environmental changes for population-level impact. As experienced in high-income market economies, addressing the structural drivers of illness and poor health will be key, including promotion and pricing of tobacco, alcohol, and unhealthy foods; air pollution; and the structures of urban living and sedentary jobs (limited physical activity opportunities and leisure/family time). Intensified intersectoral collaboration will be needed to address these drivers [82]. Increasing demands for NCD care, especially in the rapidly aging period, will pose an additional challenge for effective UHC if equitable and sustainable financing mechanisms are not improved in the near future, including cost containment for clinical services. Further investments in public transport, clean energy, and public sports/leisure facilities will be also critically important in achieving health-related SDGs in China. Finally, explicit attention to redressing intracountry and gender disparities disguised by aggregate national progress will be needed to ensure that no one is left behind and that meeting the SDGs is meaningful for all of China’s citizens.

## Supporting information

S1 Appendix Detailed methods used for the study. (PDF)

S2 Appendix Interview guidelines. (PDF)

S3 Appendix Informed consent form. (PDF)

S1 STROBE checklist STROBE, strengthening the reporting of observational studies in Epidemiology. (PDF)

## Abbreviations

BCG: Bacille Calmette-Guerin
GBD: Global Burden of Disease
GF: Global Fund
HBV: hepatitis B virus
HCV: hepatitis C virus
MCHRH: maternal and child health and reproductive health
MDG: Millennium Development Goal
MDR-TB: multidrug-resistant TB
MMR: maternal mortality ratio
NCD: noncommunicable disease
NMR: neonatal mortality rate
NRCMS: new rural cooperative medical scheme
OOP: out-of-pocket payment
PM: particulate matter
SDG: Sustainable Development Goal
SEV: summary exposure value
TB: tuberculosis
Tdap: tetanus–diphtheria–acellular pertussis
THE: total health expenditure
TRIPS: trade-related aspects of intellectual property rights
U5MR: under-5 mortality rate
UEBHI: urban employee basic health insurance
UHC: universal health coverage
UN: United Nations
WaSH: water, sanitation, and hygiene
WHO: World Health Organization
