# Supplementary material for: Current situation and progress toward the 2030 health-related Sustainable Development Goals in China: A systematic analysis
Source: PLoS Med. 2019 Nov 19;16(11):e1002975. doi: 10.1371/journal.pmed.1002975 (PMC7340487; doi:10.1371/journal.pmed.1002975)
Supplement: S2 Appendix — (PDF) [file pmed.1002975.s002.pdf]

Current Situation and Progress towards the 2030 Health-Related Sustainable Development Goals in China: A Systematic Analysis

Supporting Information File 2

Appendix - Interview Guidelines

儿童营养专题

国际组织官员访谈提纲

1. 在千年发展计划期间，中国妇女和儿童营养状况总体有什么变化趋势？她们目前面临的主要营养问题是什么？
2. 与其他发展中国家相比，中国在实现有关妇女儿童营养方面的千年发展目标中，有哪些经验和教训？
3. 对于改善妇女儿童的营养状况，贵组织有什么相关的经验呢？主要的工作重点是什么？
4. 为改善妇女儿童的营养状况，中国开展了哪些富有成效的营养干预项目？请简要介绍由 YOUR ORGANIZATION 合作开展的一些相关项目。在这些项目实施过程中（在与中国政府或当地政府合作的过程中），有没有遇到什么困难或阻力？是如何解决的？
5. 在制定千年发展目标后，世界卫生组织联合贵组织及其他相关的联合国下属组织与中国政府开展了一些相关的促进妇女儿童健康的项目，比如"改善中国最弱势妇女和儿童群体的营养、食品安全和食品保障状况项目（CFSN）"（2009-2013），还有类似这样的项目吗？请您简单介绍一下。这样的项目取得的效果如何？在实施的过程中有没有遇到什么障碍？这些项目对接下来为实现 SDG 要做的工作有什么启示？
6. 对于儿童超重和肥胖，贵组织有实施什么相关的改善措施和干预手段吗？请简单介绍一下。
7. 您如何评价目前中国的纯母乳喂养情况？在推进母乳喂养的过程中，贵组织有什么样的经验？
8. 您认为在促进中国妇女儿童营养的过程中，类似贵组织这样的国际组织在其中担任了什么样的角色？
9. 您认为目前中国距离妇女儿童营养相关的可持续发展目标还存在哪些差距？面临的最大阻碍或困难是什么？您认为中国政府可以采取哪些策略或措施来促进妇女儿童营养相关的可持续发展目标的实现？
10. 您对于我们所谈的还有什么补充？

Child Nutrition

Interview Outline for International Organization Officials

(TRANSLATION)

1. What are the overall trends in the nutritional status of Chinese women and children during the Millennium Development Goal era? What are the main nutritional problems they are currently facing?
2. What are the experiences and lessons of China in achieving the Millennium Development Goals on nutrition for women and children compared to other developing countries?
3. What are the relevant experiences of your organization to improve the nutritional status of women and children? What is the main focus of work?
4. What effective nutrition intervention projects have China carried out to improve the nutritional status of women and children? Please briefly introduce some related projects carried out by your organization. Are there any difficulties or obstacles in the implementation of these projects (in the process of cooperation with the Chinese government or local government)? How was it resolved?
5. Following the development of the Millennium Development Goals, the World Health Organization, in conjunction with your organization and other relevant United Nations organizations, has initiated projects related to the promotion of women's and children's health with the Chinese government, such as "improving nutrition and food for the most vulnerable women and children in China, safety and food security status of the project (CFSN) " (2009-2013). Are there any other projects similar to it? Please give us a brief introduction. What is the effect of such a project? Are there any obstacles encountered in the implementation process? What are the implications of these projects for the work to be done to achieve SDG?
6. What are the relevant improvement measures and interventions for your organization for children who are overweight and obese? Please give a brief introduction.
7. How do you evaluate the current exclusive breastfeeding situation in China? What kind of experience does your organization have in promoting breastfeeding?
8. What role do you think an international organization like your organization has played in promoting the nutrition of Chinese women and children?
9. What gaps do you think China is facing in achieving sustainable development goals related to women's and children's nutrition? What is the biggest obstacle or difficulty? What strategies or measures do you think the Chinese government can adopt to promote the achievement of nutrition-related sustainable development goals for women and children?
10. Are there any other thoughts you want to share?

妇幼保健

省级相关领导访谈提纲

1) 目前我省的儿童营养健康状况如何？请您概括一下，可以从生长迟缓、消瘦、低体重三个指标以及贫血、肥胖等营养性疾病方面评价。（最近两年的妇幼年报数据中5岁以下儿童营养相关指标是否可以提供？）

2) 从2000年至今，省内实施了哪些重要的儿童营养健康改善项目？您所在单位主要参与了哪些儿童营养相关的工作？有哪些值得分享的经验？在这些干预或政策实施过程中，有没有遇到什么困难或阻力？是如何解决的呢？

3) 在本省促进儿童营养状况的过程中，工作重点是什么？面对儿童肥胖与消除儿童营养不良的双重负担，省内的情况如何？您认为在本省内有什么优势或者策略可以缓解这些问题呢？

4) 请您分析一下省内纯母乳喂养的情况，近十年来纯母乳喂养率的变化趋势如何？城市与农村的情况有何区别？您觉得这种趋势受哪些因素影响呢？

5) 联合国开发计划署在2015年提出了可持续发展计划，其中包括消除一切形式的营养不良，到2025年实现5岁以下儿童生长迟缓、消瘦及营养过剩问题相关国际目标，具体而言是：1）5岁以下儿童生长迟缓比上一阶段下降40%，2）5岁以下消瘦率小于5%，3）纯母乳喂养率超过50%，4）儿童肥胖率不增加。您认为本省的情况离这些目标还有什么差距？哪些比较容易实现，而哪些是挑战或难点？如果要实现这些目标，营养改善的重点目标人群有哪些呢？您在营养改善的手段和措施方面，有什么建议呢？

Maternal and Child Health

Interview Outline for Provincial Officials

(TRANSLATION)

1) What is the current nutritional status of children in your province? Please summarize, you can evaluate the three indicators of stunting, wasting, low body weight and nutritional diseases such as anemia and obesity. (Can you help provide data of children nutrition indicators under age 5 in the annual reports published in the recent two years?)

2) What important child nutrition health improvement projects have been implemented in the province since 2000? What kind of child nutrition-related work is your institution/department involved in? What are the experiences worth sharing? Are there any difficulties or obstacles in the implementation of these interventions or policies? How to solve them?

3) What is the focus of work in promoting the nutritional status of children in the province? What is the situation in your Province facing the double burden of childhood obesity and the elimination of child malnutrition? What advantages or strategies do you think are helpful in the province to solve them?

4) How does the rate of exclusive breastfeeding change in the recent ten years in your province? What is the difference between urban and rural conditions? What factors do you think this trend is affected by?

5) The United Nations Development Program put forward a sustainable development plan in 2015, which includes the elimination of all forms of malnutrition and achieving indicators related to stunting, wasting and overnutrition issues for children under age 5 by 2025, specifically 1) Achieve a 40% reduction in the number of children under-5 who are stunted; 2) Reduce and maintain childhood wasting to less than 5%; 3) Increase the exclusive breastfeeding rate to over 50%; 4) Ensure that there is no increase in childhood overweight. Are there any gaps in your province to achieve these targets? Where are these gaps? Which targets are easier to implement and which ones are more challenging or difficult? What are the target population to improve nutrition status? What advice do you have for the means and measures for improving nutrition?

妇幼专题

国家级 FP 工作管理者访谈提纲

1. 计划生育是我国的基本国策，自上世纪 80 年代以来我国通过采取了一系列的策略和措施，使我国妇女的避孕现用率达到了很高水平，已婚育龄妇女对避孕节育服务有很高的可及性。2013 年以来，卫生与计生合并了，2015 年生育政策改变为全面二孩，在新的形势下我国计划生育服务的提供情况如何？是否继续提供免费的计划生育服务？国家有什么新的政策和措施？
2. 新的形势对我国妇女的避孕现用率有影响吗？目前的避孕现用率如何？
3. 改革开放以来，我国人民的性观念和行为有了很大的变化，青少年的首次性行为年龄提前，未婚发生性行为和同居的现象比较普遍，他们也需要生殖健康教育、信息和计划生育服务，目前我国在这方面的现状如何？国家在为未婚、青少年人群提供避孕节育服务方面有什么政策和措施？
4. 可持续发展目标中包括生殖健康方面的内容，其中要求为所有人提供综合的性与生殖健康教育和信息，我国目前性教育的现状如何？
5. 要为所有人提供综合的性与生殖健康教育和信息，特别是青少年人群，存在哪些困难和障碍？
6. 可持续发展目标中包括生殖健康方面的内容，也要求为所有人提供综合的性与生殖健康服务，我国目前的现状如何
7. 要为所有人提供综合的性与生殖健康服务，存在哪些困难和障碍？
8. 如何才能克服这些困难与挑战？
9. 您对我国实现可持续发展目标中的生殖健康指标有什么意见和建议？

MCHRH

Interview Outline for National Family Planning (FP) Administrators

(TRANSLATION)

1. Family planning is China 's basic national policy. Since the 1980s China has adopted a series of strategies and measures to increase the contraceptive use rate of women in China. Married women of childbearing age have good access to contraceptive services. The Ministry of Health and of Family Planning have merged since 2013. In 2015, the one-child policy was abolished and the two-child policy issued. What does the provision of family planning services look like in China now? Do you continue to provide free family planning services? What new policies and measures does the country have?
2. Do the new policies have an impact on the current rate of contraception for women in China? What is the current rate of contraception?
3. Since the reform and opening up, the sexual attitudes and behaviors of our people have changed dramatically. The age of experiencing the first sexual behavior among adolescents is advanced, and the phenomenon of unmarried sexual intercourse and cohabitation is more common. They also need reproductive health education, information and family planning services. What is the current status of China in this regard? What policies and measures does the country have in providing contraceptive services for unmarried and adolescents?
4. The Sustainable Development Goals include reproductive health aspects that require comprehensive sexual and reproductive health education and information for all. What is the current state of sex education in our country?
5. What are the difficulties and obstacles to providing comprehensive sexual and reproductive health education and information for all, especially for young people?
6. The sustainable development goals include reproductive health and the need to provide comprehensive sexual and reproductive health services for all, how is the current status of our country?
7. What are the difficulties and obstacles to providing comprehensive sexual and reproductive health services for all?
8. How can we overcome these difficulties and challenges?
9. What opinions and suggestions do you have for China's reproductive health in achieving the Sustainable Development Goals?

省级 FP 服务提供者访谈提纲

1. 您在 2013 年以前在什么单位工作，主要做什么工作？目前在什么单位？具体工作是什么？
2. 2013 年以来，卫生与计生合并了，2015 年生育政策改变为全面二孩，在新的形势下本省计划生育服务的提供情况如何？是否继续提供免费的计划生育服务？目前有什么新的政策和措施？
3. 本省的计划生育服务站与卫生系统合并了吗？如何合并的？已经合并的计划生育服务站开展什么工作？
4. 本省群众目前对避孕服务的需求如何？新的形势对本省妇女的避孕现用率有影响吗？目前的避孕现用率如何？主要采取什么避孕措施？
5. 改革开放以来，我国人民的性观念和行为有了很大的变化，青少年的首次性行为年龄提前，未婚发生性行为和同居的现象比较普遍，他们也需要生殖健康教育、信息和计划生育服务，目前本省在这方面的现状如何？本省在为未婚、青少年人群提供避孕节育服务方面有什么政策和措施？向未婚人群提供免费的避孕节育服务吗？
6. 可持续发展目标中包括生殖健康方面的内容，其中要求为所有人提供综合的性与生殖健康教育和信息，据您所知，本省目前性教育的现状如何？
7. 要为所有人提供综合的性与生殖健康教育和信息，特别是青少年人群，存在哪些困难和障碍？

Interview Outline for Provincial FP Service Providers

(TRANSLATION)

1. Which department/institution did you work in before 2013, and your job responsibilities? Which department/institution are you currently in and your job responsibilities?
2. The Ministry of Health and of Family Planning have merged since 2013. In 2015, the birth policy was changed from one-child to two-child policy. What is the provision of family planning services in your province now? Do you continue to provide free family planning services? Are there any new policies and measures?
3. Has the family planning service station of your province merged with the health system? How? What is the responsibility of the merged family planning service station?
4. What is the current demand for contraceptive services in your province? Do the new policies have an impact on the contraceptive use rate of women in your provinces? What is the current rate of contraception? What are the main contraceptive measures?
5. Since the reform and opening up, the sexual attitudes and behaviors of our people have changed dramatically. The age of experiencing the first sexual behavior among adolescents is advanced, and the phenomenon of unmarried sexual intercourse and cohabitation is more common. They also need reproductive health education, information and family planning services. What is the current status of your province in this regard? What policies and measures does your province have in providing contraceptive services for unmarried and adolescents? Is there a free contraceptive birth control service for unmarried people?
6. The Sustainable Development Goals include reproductive health aspects that require comprehensive sexual and reproductive health education and information for all. As far as you know, what is the current status of sex education in the province?
7. What are the difficulties and obstacles to providing comprehensive sexual and reproductive health education and information for all, especially for young people?

结核专题

国家级/省级结核病防控领导访谈提纲

1. 疫情

1）近 15 年来，我国/省结核病的疫情有什么变化、趋势和特征？　（提示：发病率、患病率、变化趋势、区域差异、人群差异、重点人群）

2）您如何评价近年来结核病防控的效果？有哪些问题？（提示：发病率总体下降较慢，在部分西部和农村地区略有上升是为什么？）

3）近 15 年来，我国/省耐多药结核病的疫情有什么变化和趋势？

4）您如何评价近年来耐多药结核病防控的效果？有哪些问题？

2. 肺结核防治策略与体系

1）策略: 能否请您简要概括下近 15 年来主要的结核防治策略（政策、项目）？

2）服务体系：请您简要介绍下目前结核病防治体系。主要存在哪些问题（提示：定点医院、疾控中心、基层的问题；经费、人员、协调、能力、保障等方面的问题）？有哪些成功经验？

3. 患者发现、治疗、管理与疫情监测

1）您认为目前患者发现、治疗和管理如何？存在哪些问题？

2）定点医院的诊疗存在哪些问题？

3）患者管理存在哪些问题？

4）目前新诊断技术和新药品的使用情况如何？是否有推广计划？

5）您认为如何提高患者发现率、改善治疗和管理？

6）您认为现在结核病疫情监测与报告存在什么问题？如何改善？

4. 筹资与保障体系

1）筹资：我国/省目前肺结核防治的经费主要有哪些（类别、来源、大致金额、分配与使用情况）？（提示：基本公卫、医保、专项、医疗救助）

2）经费：我国/省结核防治经费是否足够？如有缺口，缺口有多少？

3）经费：您觉得应该如何筹资以保证对结核的投入？

4）负担：您觉得结核病患者经济负担如何？

5）负担：您认为造成患者经济负担的原因是什么？

6）您认为可以采取哪些措施降低患者的经济负担？

5. 未来工作规划

1）对未来 5 年和 15 年内的结核病防治有什么样的规划？哪些工作重点？哪些难点？

2） 对未来 5 年和 15 年内的耐多药结核病防治有什么样的规划？哪些工作重点？哪些难点？

6. SDG 和健康中国 2030 目标的实现

1） 联合国可持续发展目标和健康中国２０３０对结核病防控制定了总目标和阶段性目标，各省也根据本地情况制定了目标【访谈人描述具体目标】。您认为这些目标是否能达到？

2） 您认为达到目标有哪些困难？

3） 为了达到目标，应该采取何种策略和措施？

4） 是否有相关的测算？

7. 经验与建议

1） 您对实现结核病相关的健康中国２０３０和可持续发展目标有何建议？

2） 您对我们的研究有何建议？您认为什么样的研究有助于政策的制定？

Tuberculosis

Interview Outline for TB Prevention and Control Officials at National/Provincial Level

(TRANSLATION)

1. Epidemic
    1) What are the epidemiological changes, trends and characteristics of tuberculosis in China/the province in the past 15 years? (Hint: incidence, prevalence, trends, regional differences, population differences, key populations)
    2) How do you evaluate the effectiveness of tuberculosis prevention and control in recent years? What are the problems/challenges? (Hint: The overall decline in the incidence rate is slow, and why is there a slight increase in some western and rural areas?)
    3) What are the epidemiological changes and trends in the epidemic of multidrug-resistant tuberculosis in China/the province in the past 15 years?
    4) How do you evaluate the effectiveness of multidrug-resistant tuberculosis prevention and control in recent years? What are the problems/challenges?
2. Tuberculosis prevention and control strategy and system
    1) Strategy: Can you briefly summarize the main TB prevention and control strategies (policies, projects) in the past 15 years?
    2) Service Delivery System: Please briefly introduce the current tuberculosis prevention and treatment system. What are the main problems/challenges (Hint: designated hospitals, CDCs, issues at the primary level; funding, personnel, coordination, capabilities, security, etc.)? What are the successful experiences?
3. Patient identification, treatment, management, and epidemic monitoring
    1) What do you think of current patient identification, treatment, and management? What are the problems?
    2) What are the problems in the diagnosis and treatment of designated hospitals?
    3) What are the problems with patient management?
    4) What does the current uptake of new diagnostic techniques and new drugs look like? Is there a promotion plan?
    5) How do you think about improving patient identification, improving treatment and management?
    6) What problems do you think exist in the current monitoring and reporting of tuberculosis? how to improve?
4. Financing and insurance system
    1) Financing: What are the main funding sources for the prevention and treatment of tuberculosis in China/the province (category, source, approximate amount, distribution and use)? (Hint: basic public health, medical insurance, special fund, medical assistance)

2) Funding: Is the funding for tuberculosis prevention and control in China/the province sufficient? If there is a gap, how big is it?

3) Funding: How should we finance to ensure the investment in tuberculosis control?

4) Burden: What do you think of the economic burden of TB patients?

5) Burden: What do you think is the cause of the patient's financial burden?

6) What measures can you take to reduce the financial burden on patients?

5. Future work planning

   1) What kind of planning is there for tuberculosis prevention in the next 5 and 15 years? What is the main focus of work? What are the difficulties?

   2) What kind of planning is there for MDR-TB prevention in the next 5 and 15 years? What is the main focus of work? What are the difficulties?

6. Implementation of SDG and Healthy China 2030 Target

   1) The UN Sustainable Development Goals and Healthy China 2030 set general goals and milestones for tuberculosis prevention and control. The provinces also set targets based on local conditions [interviewers describe specific targets]. Do you think these goals can be achieved?

   2) What difficulties do you think are reaching the goals?

   3) What strategies and measures should be taken to achieve the goals?

   4) Is there any forecasting/projection available?

7. Experience and recommendations

   1) What advice do you have for achieving TB-related Healthy China 2030 and the Sustainable Development Goals?

   2) What advice do you have for our research? What kind of research do you think can contribute to policy development?

慢病专题

国家慢病防控领导访谈提纲

1. 慢性病流行现状与发展趋势

（1）我国目前慢性病的流行特征，近 20 年来我国慢性病的流行情况是如何变化的？整体趋势如何？（患病率与死亡率，有无明显的区域差异、人群差异）

（2）近五年来，我国慢病防控的工作成效如何？（典型的成功案例？）我国目前的慢病防控工作现况如何？存在的主要问题是什么？

2. 慢性病防控政策体系

（1）您认为现在的慢病防控策略能否满足现在的慢病形势？为了降低慢病导致的过早死亡，我国近年来有什么样的举措、在政策上有了哪些变动？

（2）就我国慢病防控工作现况来看，您认为促进医防协同落实医防结合主要面临哪些问题？有什么解决办法？

（3）全国慢病综合防控示范区建设的效果如何？其成功经验现能否在更多地区推广？推广中将面临的最大挑战是什么？

3. 经费与保障

（1）我国投入慢病防控的资金占国家卫生经费的比例？目前慢性病防控资金的筹资渠道主要有几种？您认为还有可以增加的渠道吗？

（2）目前我国慢病防治资金中预防经费的比例远小于治疗经费，您认为可以采取什么措施来改变这种情况？

（3）为了与慢病防控工作有机结合，切实减轻慢病患者负担，您认为我国现行的医保政策和救助政策有哪些方面需要改革？

4. 人力资源

（1）目前我国从事慢性病防控的工作人员数与我国慢病防控工作需要是否匹配？分布是否平衡?工作人员的专业素养与业务能力是否适应工作需要(质量如何)？

（2）就我国慢病防控现状看来，您认为我国慢病防控领域的人员亟需具备何种人才？如何能引导人才流向基层，流向社区？

5. 关于 SDG 和健康中国 2030 目标的实现

（1）对于可持续发展目标和健康中国 2030 中有关慢性病的指标，您认为是否可以实现？实现这些目标最大的困难在哪里？为实现目标，我国下一步慢病防控工作的重点是什么？

（2）对于将非传染系疾病导致的过早死亡降低三分之一这一目标，国家或省级有没有做相应的经济学测算？（或者在降低慢病导致的过早死亡方面，国家有没有相应的成本测算？）

（3）对于今年颁布的《中国防治慢性病中长期规划(2017-2025 年)》，该规划与健康中国2030目标有什么联系？您认为其中的八项策略措施能否保障实现健康中国2030目标和 SDG 目标？

6. 建议

（1）您对加强慢病防控工作、实现 SDG 和健康中国有关慢病防控目标有何建议？（可提示：有无必要专门立法？实现全流程健康管理的关键？可以动员那些社会力量？）

（2）为了支持慢性病防控工作，您认为今后应着重哪些方面的科学研究与探索？

控烟相关领导访谈提纲

1. SDG 慢病目标是否合理、有无可能达到？
2. 目前控烟的有效措施和面临的挑战，如何解决？
3. 中国加入公约后的努力方向和政策导向？如何加强？
4. 怎么理解控烟和反控烟的博弈？这种状况需要如何改变？
5. 控烟和慢病防治的结合现状？存在的问题，解决的办法？

省级慢病防控领导访谈提纲

1. 本省慢病现状、发展趋势、控制重点
    1) 您如何评价本省近 20 年来慢病的现状和发展趋势？
    2) 结合健康中国 2030，下一步本省慢性病的控制重点是什么？
2. 本省预防体系的现状及存在的问题
    1) 您能否简略的描述本省近 20 年来预防慢性病（各重点疾病）的主要预防策略（政策、项目）？
    2) 您认为这些预防策略、项目效果如何？有什么问题（提示：政策制定者、疾控中心、基层的问题；经费、人员、协调、能力等方面的问题）？有哪些成功的经验和解决的方法？
3. 本省慢病控制的具体实施方案
    1) 本省距健康中国 2030 目标有多大的距离？
    2) 是否制定本省的健康 2030 目标？
    3) 本省有何健康考核指标？
    4) 本省控制慢性病的具体实施方案是什么？

4.国家对慢病的支持力度及需要

    1) 国家对本省控制慢性病的支持力度如何？
    2) 本省在控制慢性病方面还有什么需要？（人力、物力）

NCDs

Interview Outline for NCDs Prevention and Control Officials at National Level

(TRANSLATION)

1. Current status and development trend of NCDs

    1) Regarding the epidemiological characteristics of NCDs in China, how has the prevalence of NCDs changed in China in the past 20 years? What is the overall trend? (prevalence and mortality; whether there are obvious regional differences, population differences)

    2) In the past five years, how has the effectiveness of prevention and control of NCDs in China been achieved? (Typical success stories?) What is the current status of prevention and control of NCDs in China? What are the main problems?

2. NCDs prevention and control system

    1) Do you think the current NCDs prevention and control strategy can control the current NCDs epidemic? In order to reduce the premature death caused by NCDs, what kind of measures has China made in recent years, and what changes have been made in terms of control policies?

    2) Judging from the current NCDs control work conducted, what are the main problems do you think to face the promotion of medical prevention and treatment? What are the solutions?

    3) What is the effect of the construction of the national comprehensive prevention and control demonstration zone for NCDs? Can successful experience be promoted in more regions? What is the biggest challenge facing the promotion?

3. Financing and insurance

    1) What proportion of funds invested in NCDs prevention and control in China accounts for national health expenditure? At present, there are several financing channels for the prevention and control of NCDs. Do you think there are channels that can be added?

    2) At present, the proportion of prevention funds in China's NCDs prevention and control funds is far less than the treatment funds. What measures can you take to change this situation?

    3) In order to effectively combine the prevention and control of NCDs and effectively reduce the burden of NCDs patients, what aspects do you think China's current health insurance policies and assistance policies need to be reformed?

4. Human resources

    1) Does the number of health staff engaged in the prevention and control of NCDs in China match the needs of prevention and control of NCDs? Is the distribution balanced? Are they qualified in terms of professionalism and capacity (how about the quality)?

    2) Regarding the current situation of prevention and control of NCDs in China, what kind of talents do you think people in the field of NCDs prevention and control

need in China? How can we guide talents to the grassroots and to the community?

5. About achieving SDG and Healthy China 2030 goals

   1) Do you think that the NCDs-related Sustainable Development Goals and Healthy China 2030 can be achieved? What is the biggest difficulty in achieving these goals? In order to achieve the goals, what is the focus of the next step of prevention and control of NCDs in China?

   2) Is there any economic forecasting at the national or provincial level about whether we can reduce premature death from NCDs by one-third? (Or does the country have a costing projection?)

   3) What are the relationships between Healthy China 2030 and the recently issued "China's Long-term Plan on Prevention and Control of NCDs (2017-2025 years)"? Do you think that the eight strategic measures can help achieve the healthy China 2030 target and the SDGs?

6. Suggestions

   1) What suggestions do you have for strengthening the prevention and control of NCDs, achieving NCDs-related SDG and Healthy China 2030 goals? (Hint: Is there a need for special legislation? The key to achieving full-process health management? Can you mobilize the social forces?)

   2) In order to support the prevention and control of NCDs, what areas of scientific research and exploration do you think should be emphasized in the future?

Interview Outline for National Tobacco Control Officials

1. Is the NCDs-related SDG target reasonable and achievable?

2. What are the effective measures and challenges of tobacco control? How to solve the challenges?

3. China's efforts and policy orientation after joining the Convention? How to strengthen them?

4. How to understand the game of tobacco control and counter-control? How does this situation need to change?

5. What is the current status of integrating tobacco control and NCDs prevention? Problems, solutions?

Interview Outline for NCDs Prevention and Control Officials at Provincial Level

1. The current situation, development trend, and control focus of NCDs in the province

   1) How do you evaluate the current epidemiological situation and development trend of NCDs in the province in the past 20 years?

   2) Combining healthy China 2030, what is the focus of control of NCDs in the province?

2. The status quo and existing problems of the province's prevention system

1) Can you briefly describe the main prevention strategies (policies, projects) for preventing NCDs (high priority diseases) in the past 20 years?

2) What do you think of these prevention strategies and projects? What are the problems (Hint: policy makers, CDCs, issues at the primary level; funding, personnel, coordination, capabilities, etc.)? What are the successful experiences and solutions?

3. The specific implementation plan of NCDs control in this province

3) How far is the province to achieve the NCDs-related Healthy China 2030 Goals?

4) Has the province set up its own 2030 target on NCDs?

5) What health indicators are there in the province?

6) What is the specific implementation plan for controlling NCDs in the province?

4. National support for NCDs and needs

7) What is the state's support for the province's control of NCDs?

8) What are the needs of the province in controlling NCDs? (human, material, etc.)
