# Supplementary material for: Current situation and progress toward the 2030 health-related Sustainable Development Goals in China: A systematic analysis
Source: PLoS Med. 2019 Nov 19;16(11):e1002975. doi: 10.1371/journal.pmed.1002975 (PMC7340487; doi:10.1371/journal.pmed.1002975)
Supplement: S3 Appendix — (PDF) [file pmed.1002975.s003.pdf]

Current Situation and Progress towards the 2030 Health-Related Sustainable Development Goals in China: A Systematic Analysis

Supporting Information File 3

Appendix - Informed Consent Form

Informed consent for Key Informants

(TRANSLATION)

Introduction:

Hello, my name is ________________. I am working with Dr. Shenglan Tang of Duke University in the United States to conduct research on Achieving Health SDGs in China: Developing Evidence-based Policy Options for Action. The study is sponsored by the Bill & Melinda Gates Foundation. **All information collected will only be used for research purpose.**

Research studies are voluntary. As I read this form to you, please take your time deciding whether to participate. Please ask me to explain anything that you do not clearly understand. The purpose of the study, procedures, risks, and benefits are described below.
The research team will give you a copy of this form. It is important that you know:

- Your participation is entirely voluntary;
- You may decide not to take part or to withdraw from the study at any time

Study purpose:

The purpose of this study is to understand the current situation, achievements, and main challenges in achieving health related SDGs.

Who Will Be in This Study and How Long Will This Study Last?

A number of relevant stakeholders will participate in this study overall. We will have one-on-one interview with officials from government including: Health and Family Planning Commission (HFPC), Center of Disease Control (CDC), Human Resources and Social Security (HRSS), Heads hospitals, Civil Affairs, and Finance. Each key informant interview will last approximately 60-90 minutes. Your participation in the study will end after the discussion ends.

Procedures:

If you choose to participate in the study, we will ask you a series of questions related to your role and your opinion about achieving SDGs in China. Your responses will be

audio recorded. If you do not want to be recorded, please let me know. Audio-recording is just a back-up for note-taking.

Benefits:

There are no direct benefits to you from this study. However, the information that you provide will help us to improve China's strategy in achieving health related SDGs.

Confidentiality:

Your personal information, such as name, address or other identifying information about you and your families will not be noted or recorded. The audio recordings of the interview will begin after the brief self-introduction at the beginning of the interview.
All identifying information collected from you will be kept confidential and accessed only by authorized personnel associated with this study. You will not be identified personally in any publications resulting from this study.
Authorized personnel (Duke University, Duke Kunshan University, Wuhan University, Fudan University, Kunming Medical University, Chongqing Medical University, DRC and NHDRC) **are the investigators of this study, who must sign the agreement of confidentiality and receive IRB training before the interview.**

Voluntary participation/right to withdraw:

Participation in this study is voluntary. You may choose to discontinue your participation at any time during the study. The information you provide to us will be used even if you choose to discontinue your participation. However, no new information will be collected once you discontinue from the study.

Whom do I call if I have questions or problems?

For questions about this study or if you have problems, concerns, questions, or suggestions about the research, contact Dr. Shenglan Tang at Duke University (telephone number 1-(919)- 681-8857 or Dr. Xiaohua Ying in Fudan University of China (telephone number 86-(21)- 54237283.
If you decide to withdraw, please feel free to inform the investigator at any time and we will immediately stop the interview.
For questions about your rights as a research participant or to discuss problems or concerns related to the research, contact the Duke University Ethics Committee at 919-684-3030 and campusirb@duke.edu

VOLUNTEER AGREEMENT

Check box if participant agrees to audio-recording

I certify that the nature and purpose, the potential benefits, and possible risks associated with participating in this research have been explained to me and that informed consent was obtained.

*To protect the confidentiality of interviewees, the verbal consent is required and no signature is needed.
