# Supplementary material for: Current situation and progress toward the 2030 health-related Sustainable Development Goals in China: A systematic analysis
Source: PLoS Med. 2019 Nov 19;16(11):e1002975. doi: 10.1371/journal.pmed.1002975 (PMC7340487; doi:10.1371/journal.pmed.1002975)
Supplement: S1 STROBE checklist — (PDF) [file pmed.1002975.s004.pdf]

Current Situation and Progress towards the 2030 Health-Related Sustainable Development Goals in China: A Systematic Analysis

Supporting Information File 1

STROBE Checklist

STROBE Statement

Current Situation and Progress towards the 2030 Health-Related Sustainable Development Goals in China:

A Systematic Analysis (China SDG study)

	Item No.	Recommendations	China SDG Study
Title and abstract	1	(a) Indicate the study's design with a commonly used term in the title or the abstract;	The study design "a systematic analysis" is contained in the title.
		(b) Provide in the abstract an informative and balanced summary of what was done	The abstract summarizes the background, methods and findings and conclusions of the study.
Introduction			
Background/rationale	2	Explain the scientific background and rationale for the investigation being reported	The five paragraphs of the Introduction well explain the scientific background and rational for this study.
Objectives	3	State specific objectives, including any prespecified hypotheses	The objectives can be found in the last paragraph of the Introduction session.
Methods			
Study design	4	Present key elements of study design early in the paper	The key elements of study design are presented in the first and second sub-section of the Methods.
Setting	5	Describe the setting, locations, and relevant dates, including periods of recruitment, exposure, follow-up, and data collection	They are described in the first sub-section of the Methods. Note that the study uses secondary panel data (group level) for analysis and therefore does not involve recruitment, follow-up etc.

Participants	6	(a) Cohort study—Give the eligibility criteria, and the sources and methods of selection of participants. Describe methods of follow-up Case-control study—Give the eligibility criteria, and the sources and methods of case ascertainment and control selection. Give the rationale for the choice of cases and controls<br>Cross-sectional study—Give the eligibility criteria, and the sources and methods of selection of participants	The study uses secondary panel data (group level) for analysis and therefore does not involve selection of participants for quantitative data collection.<br><br>The selection of key informants for qualitative data collection is descried in the second paragraph of the first sub-section, Sources of Data, of the Methods.
		(b) Cohort study—For matched studies, give matching criteria and number of exposed and unexposed Case-control study—For matched studies, give matching criteria and the number of controls per case	N/A
Variables	7	Clearly define all outcomes, exposures, predictors, potential confounders, and effect modifiers. Give diagnostic criteria, if applicable	The key variables of the study are the SDG indicators and the attainment index which are described in the second, third and fourth sub-section of the Methods.
Data sources/ measurement	8	For each variable of interest, give sources of data and details of methods of assessment (measurement). Describe comparability of assessment methods if there is more than one group	Descried in the first, second and last sub-section of the Methods.
Bias	9	Describe any efforts to address potential sources of bias	Descried in the last sub-section, Data quality and methodology robustness, of the Methods.
Study size	10	Explain how the study size was arrived at	N/A
Quantitative variables	11	Explain how quantitative variables were handled in the analyses. If applicable, describe which groupings were chosen and why	Explained in the second, third, fourth and fifth sub-section of the Methods.
Statistical methods	12	(a) Describe all statistical methods, including those used to control for confounding	Described the projection methods in the third sub-section of the Methods and appendix.
		(b) Describe any methods used to examine subgroups and interactions	N/A
		(c) Explain how missing data were addressed	N/A
		(d) Cohort study—If applicable, explain how loss to follow-up was addressed Case-control study—If applicable, explain how matching of cases and controls was addressed	N/A

		Cross-sectional study—If applicable, describe analytical methods taking account of sampling strategy	
		(e) Describe any sensitivity analyses	N/A
Results			
Participants	13	(a) Report numbers of individuals at each stage of study—eg numbers potentially eligible, examined for eligibility, confirmed eligible, included in the study, completing follow-up, and analysed	N/A
		(b) Give reasons for non-participation at each stage	N/A
		(c) Consider use of a flow diagram	N/A
Descriptive data	14	(a) Give characteristics of study participants (eg demographic, clinical, social) and information on exposures and potential confounders	N/A
		(b) Indicate number of participants with missing data for each variable of interest	
		(c) Cohort study—Summarise follow-up time (eg, average and total amount)	
Outcome data	15	Cohort study—Report numbers of outcome events or summary measures over time	The SDG indicators are presented in Table 1.
		Case-control study—Report numbers in each exposure category, or summary measures of exposure	
		Cross-sectional study—Report numbers of outcome events or summary measures	
Main results	16	(a) Give unadjusted estimates and, if applicable, confounder-adjusted estimates and their precision (eg, 95% confidence interval). Make clear which confounders were adjusted for and why they were included	Presented in Table 2, 3 and Figure 1 and 2 and throughout the results section by seven thematic topics.
		(b) Report category boundaries when continuous variables were categorized	N/A
		(c) If relevant, consider translating estimates of relative risk into absolute risk for a meaningful time period	N/A

Other analyses	17	Report other analyses done—eg analyses of subgroups and interactions, and sensitivity analyses	N/A
Discussion			
Key results	18	Summarise key results with reference to study objectives	Summarized in the first sub-section of the Discussion.
Limitations	19	Discuss limitations of the study, taking into account sources of potential bias or imprecision. Discuss both direction and magnitude of any potential bias	Discussed in paragraph 2-7 of the third sub-section of the Discussion.
Interpretation	20	Give a cautious overall interpretation of results considering objectives, limitations, multiplicity of analyses, results from similar studies, and other relevant evidence	Interpreted in the fourth and fifth sub-section of the Discussion.
Generalizability	21	Discuss the generalisability (external validity) of the study results	N/A
Other information			
Funding	22	Give the source of funding and the role of the funders for the present study and, if applicable, for the original study on which the present article is based	Presented in the third paragraph of the Acknowledgement.
